# Inhibitory effects of cadmium and hydrophilic cadmium telluride quantum dots on the white rot fungus *Phanerochaete velutina*

**DOI:** 10.1016/j.heliyon.2024.e41190

**Published:** 2024-12-12

**Authors:** Florian Part, Christian Zafiu, Eva-Kathrin Ehmoser, Mika A. Kähkönen

**Affiliations:** a BOKU University, Department of Water-Atmosphere-Environment, Institute of Waste Management and Circularity, Muthgasse 107, 1190, Vienna, Austria; b BOKU University, Department of Bionanosciences, Institute of Synthetic Bioarchitectures, Muthgasse 11, 1190, Vienna, Austria; c Department of Microbiology (Biocenter 1, Viikinkaari 9), Faculty of Agriculture and Forestry, University of Helsinki, Finland

**Keywords:** Quantum dots, Particle coating, White rot fungus, Decolorization, Cadmium, Reactive Black 5, Nano

## Abstract

The white rot fungus *P. velutina* was investigated for its ability to decolorize the reactive textile dye Reactive Black 5 (RB5) that was co-exposed to CdCl_2_ and quantum dots (QDs) consisting of a CdTe core capped with two different hydrophilic organic ligands (NAC and MPA). Without co-exposure, *P. velutina* completely decolorizes RB5 within 9 days. The highest inhibitory effect was found for soluble CdCl_2_ with an EC_50_ of 583 μg l^−1^, followed by MPA-QDs (10,628 μg l^−1^) and NAC-QDs (17,575 μg l^−1^). The different EC_50_ values indicate that the nanoparticle coatings have a strong influence on the inhibitory effects, as the organic ligands used for surface passivation prevented the leaching of acute toxic Cd ions from the metallic QD core. Compared to CdCl_2_, the CdTe QDs were less inhibitory to the formation of fungal metabolites and their decolorization ability on co-exposed textile dyes. In addition, the literature comparison suggests that *P. velutina* is more resilient to cadmium than other microorganisms. The fungus could therefore be used for bioremediation applications of dye- or QD-contaminated wastewater from the textile or semiconductor industries.

## Highlights

• *P. velutina* degrades RB5 within 9 days.
• Soluble Cd is more toxic than CdTe QDs.
• White rot fungi are more resilient towards Cd than other microorganisms.

## Graphical abstract

**Figure:**
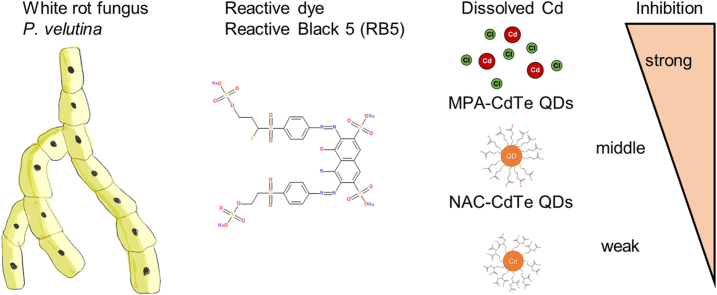

## 1 Introduction

The production volume of engineered nanomaterials will double between 2021 and 2031 [1], of which ca. 90 % will end up in landfills at the end of their useful life. More than 700′000 tons per year of nanomaterials will be applied to electronics and optics, 8000 tons per year will be used for solar panels [1]. Semiconductor quantum dots (SQDs) are used for such applications, as these small engineered nanoparticles (ENPs) – with a geometric size of a few nanometers (<10 nm) – have outstanding size-dependent optical and photoluminescence properties [[2], [3], [4]]. SQDs are used as photostable fluorescent markers for medical applications, sensorics [5,6], or for semiconductors in QD-based solar cells or QD-LED applications (e.g., for ‘QLED’ TV) [7,8]. For 2020, their production volume was estimated at up to 5 tons per year and very high growth rates are expected (58 % per year) due to renewable energy and the electronics industry [9]. With the predicted increase in production volumes, the quantities of wastewater and solid waste will also increase, and with them the environmental impact. During the production phase, QDs may end up in wastewater or solid waste streams from the semiconductor industry. Exposure during the use phase is unlikely as long as the QDs are incorporated into the product components, from where they cannot be released from the solid product matrix without high mechanical or chemical stress [[10], [11], [12], [13]]. However, QDs can be released from nano-enabled products during end-of-life processes, such as mechanical, chemical or thermal waste treatment, and as a result harm human health and the environment – for example, QD-contaminated fine dusts (particulate matter) that can be released during shredding processes [13].

For this study, a worst-case scenario was assumed where wastewater from R&D laboratories or the semiconductor industry may contain QD-containing waste with hydrophilic surface properties. These QD types are mostly used for medical research (e.g., cancer diagnostics), but also for QD-based emerging solar cells [3,14]. At the end-of-life QDs may enter the wastewater stream through washing or cleaning processes and thus contaminate effluents, which in turn must be treated in wastewater treatment plants (WWTP). In a WWTP, it can be assumed that most of the end-of-life QDs will hetero-aggregate with dissolved organic matter or other suspended particles during physical-chemical or biological wastewater treatment and thus may be removed or transferred to the sewage sludge. These sludges, in turn, are thermally or biologically further treated, or directly used as fertilizer in agriculture. The literature indicates that QDs can be persistent in aqueous environments or soils [10,15] and therefore alternative treatment strategies should be developed that enable a degradation of the end-of-life QDs. The white rot fungus *Phanerochaete velutina* showed promising properties and could serve as an alternative to treat wastewaters in which textile dyes are co-present with inorganic pollutants such as heavy metals [16]. *P. velutina*, which belongs to the basidiomycetous fungi, produce non-specific extracellular oxidative enzymes that enables the biochemical degradation of persistent organic pollutants [17]. These excreted oxidative enzymes are able to decompose even persistent natural and xenobiotic compounds [18,19]. Therefore, these fungi are explored for environmental biotechnology to bioremediate e.g., dye-containing wastewaters and contaminated soils [20,21]. However, the enzymatic performance in decolorizing dyes is also dependent on co-pollutants such as heavy metals or nanoparticles that can inhibit the process [[22], [23], [24], [25], [26]]. Given that SQDs consist of heavy metals and calcogens, inhibitory effects on fungal species appear probable in analogy to findings for silver nanoparticles [[27], [28], [29]]. However, there is only scarce information on the toxicity of SQDs and other co-pollutants on fungal species. Therefore, this study aimed to investigate toxicity and inhibitory effects on the decolorization of the synthetic dye Reactive Black 5 (RB5) with respect to two differently coated, water-dispersible QDs in comparison to dissolved cadmium ions.

## 2 Material and methods

### 2.1 Preparation and characterization of quantum dots

For this study, the same SQDs were used as reported earlier [30]. Similar to the synthesis protocols, two differently coated SQD types were produced, which particle surfaces were modified to render them hydrophilic. One SQD type consists of a CdTe particle core that was additionally capped with the organic ligand *N*-acetyl-*L*-cysteine (NAC). The second SQDs type was made in the same way, but instead of NAC, the capping agent 3-mercaptopropionsäure (MPA, Sigma-Aldrich, CAS no. 107-96-0) was used to passivate the CdTe core. These two water-dispersible QD types are referred to as “NAC-QDs” and “MPA-QDs”, respectively, for this study.

**Fig. 1:**
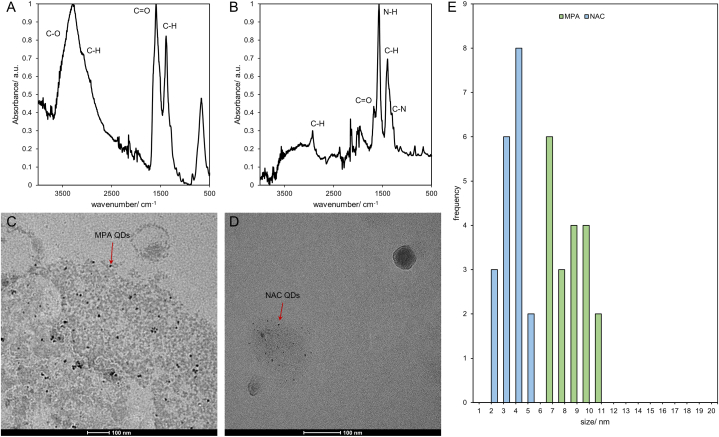
FTIR and TEM characterization of MPA- and NAC-QDs. ATR-FTIR spectra of (A) MPA-QDs and (B) NAC-QDs. Transmission electron micrographs of (C) MPA-QDs and (D) NAC-QDs, with scale bars representing 100 nm and red arrows indicating an individual QD. (E) Size-histogram (diameter) determined from Feret's diameters (N = 4 each) for 20 individual MPA-QDs (green) and NAC-QDs (blue).

The ligand exchange was analyzed by ATR-FTIR (Attenuated total reflection Fourier transform infrared) spectroscopy (Alpha, Bruker, Germany) using a DTGS (Deuterated Triglycine sulphate) detector. For analysis the aqueous QD samples (150 mg l^−1^) were transferred to the diamond ATR crystal and dried using a hot gun until the large water peaks disappeared (in the spectrum using scanning mode) until a fine and dry film was formed. Once the spectra did not change any more, the spectrum was recorded as a mean spectrum of 32 scans at a resolution of 4 cm^−1^ in a spectral range of 4000–500 cm^−1^. The reference spectrum was recorded in air at the same settings as the sample and the crystal was cleaned by 1 M HNO_3_ and ethanol. The normalized spectra (vs. the highest peak intensity) show characteristic organic bands of the ligands (Fig. 1). In case of MPA-QDs (Fig. 1 A), a broad band that consisted of two convoluted bands, O-H stretching and C-H stretching) was found in the range of 3700–2620 cm^−1^. Additionally, the C=O stretching (1700 - 1600 cm^−1^) and C-H stretch (1400 - 1200 cm^−1^) vibrations were observed. The band centered around 650 cm^−1^ could not be attributed to the ligands and the characteristic S-H band (2650–2550 cm^−1^) was not observed, which indicates that the ligands that were analyzed were bound to the QDs. In case of NAC-QDs (Fig. 1 B), the broad band was less pronounced and the C-H stretch vibration was more visible (2912 cm^−1^). A few bands found between 2150 and 1990 cm^−1^, which indicate the formation of thioisocyanate, which may be a degradation product of the ligand. This may have well happened as the IR spectra were recorded after more than 1 year after the exposure experiments were conducted. The C=O stretching vibration was found at ca. 1700 cm^−1^, as well as the dominant N-H bending (1580 cm^−1^), C-H bending (ca. 1400 cm^−1^) and a small convoluted C-N stretching band (ca. 1200 cm^−1^). Similar to the MPA-QDs, no thiol bands could be observed, which indicates that NAC was either covalently bound to the QDs or oxidized. The charge of the particle was analyzed by Zeta-potential measurements (Nanozetasizer, Malvern, UK) using folded capillary cuvettes (DTS 1070, Malvern, UK). NAC-QDs exhibited a zeta-potential of −39.4 mV (±21.2) and MPA-QDs −48.9 mV (±16.1), showing that both nanoparticles were negatively charged due to the ligands.

The size of the QDs was determined by TEM (FEI Tecnai G2 20 transmission electron microscope) operating at 160 kV. The samples were prepared by dropping aqueous dispersions (150 mg l^−1^) onto 300 mesh, Pioloform coated copper grids (Plano GmbH, Germany) that were previously activated in an ozonator and subsequently blotted by a paper filter (Fig. 1C and D, MPA- and NAC-QDs, respectively). The size was determined by determining the Feret's diameter of the particles as a mean of four measurements of each particle in ImageJ (NIH, USA). The mean value and size distribution was evaluated for 20 representatively selected QDs each (Fig. 1 E) and resulted in a mean value of 8.3 nm (±1.3) and 2.9 nm (±0.7) for MPA-QDs and NAC-QDs, respectively. The diameters determined by TEM measurements were used to determine the molar concentration of the QDs resulting in a molecular mass of 44987.23 g mol^−1^ (with a density of 5.85 g cm^−3^ and a molecular mass of 240.01 g mol^−1^ for bulk CdTe) for NAC QDs. In analogy, a molecular mass of 1054701.79 g mol^−1^ for MPA-QDs was calculated. Using these parameters allowed to calculate the molar concentration of the QDs and the Cd content as shown in Table 1, whereas the cadmium content is the same due to the same composition for both types of QDs. In addition, the fluorescence emission maxima were determined for both QDs on a fluorescence spectrometer with 500 nm for NAC-QDs and 539 nm for MPA-QDs. Further physicochemical properties of the QDs, which were determined using UV–VIS, TEM, fluorescence spectroscopy (photoluminescence quantum yield) and DLS (hydrodynamic diameters), can be found in our previous studies [10,30,31].

**Table 1:** Concentrations of Cd quantity (μM and μg l^−1^) for NAC-QDs and MPA-QDs, as well as QD particle concentration (μM QD).

Concentration	CdCl_2_	NAC-QDs			MPA-QDs		
μg l^−1^	μM	μM QD	μM Cd	μg l^−1^ (Cd)	μM QD	μM Cd	μg l^−1^ (Cd)
50	0.27	1.11·10^−3^	0.21	23.4	4.74·10^−5^	0.21	23.4
100	0.55	2.22·10^−3^	0.42	46.8	9.48·10^−5^	0.42	46.8
500	2.73	1.11·10^−2^	2.08	234	4.74·10^−4^	2.08	234
1000	5.46	2.22·10^−2^	4.17	468	9.48·10^−4^	4.17	468
2500	13.64	5.56·10^−2^	1.04	1170	2.37·10^−3^	1.04	1170
5000	27.28	1.11·10^−1^	20.8	2340	4.74·10^−3^	20.8	2340
7500	40.91	1.67·10^−1^	31.2	3510	7.11·10^−3^	31.2	3510
10,000	54.55	2.22·10^−1^	41,7	4680	9.48·10^−3^	41,7	4680
20,000	109.10	4.45·10^−1^	83.3	9370	1.90·10^−2^	83.3	90,370
30,000	163.66	6.67·10^−1^	125	14,100	2.84·10^−2^	125	14,100

### 2.2 Test cultivations

The white rot fungus *Phanerochaete velutina* (FBCC 941, previous number 244i) was obtained from the Fungal Biotechnology Culture Collection (FBCC) at the Department of Microbiology at the University of Helsinki in Finland. The basidiomycetous white rot fungus, *P. velutina*, was pre-grown on malt extract-agar (MEA) plates during seven days at 28 °C. Five MEA plugs (4 mm diameter) of pre-grown fungus from the plates were added to sterilized LN-AS medium (pH 4.5; 75 ml). The growth media had a final glucose content of 0.5 % (wt/vol) as carbon source. The liquid LN-AS medium for pre-growth incubations were made in two 250 ml flasks, which were shaken for seven days at 28 °C. *P. velutina* was well-grown in liquid solutions. Both pre-growth LN-AS liquid cultures were combined and mixed with a sterile stirrer.

### 2.3 Assay

Four different plates in different setups were prepared for each samples containing NAC-QDs, MPA-QDs or Cd as CdCl_2_ (Table 2) in concentrations of 0, 50, 100, 500, 1000, 2500, 5000, 7500, 10,000, 20,000, and 30,000 μg l^−1^. Each concentration was applied in quadruplicate to the plate. The first plate was prepared without Reactive Black 5 (Sigma-Aldrich, Germany, CAS no. 17095-24-8, referred as “RB5”) and without fungus to investigate the background absorbance of the studied materials. The second plate was prepared with RB5 and without the fungus to investigate the stability of the dye in presence of the materials. The third plate contained only fungus and the materials to investigate absorbance change that were associated with the fungus. The fourth plate contained fungi and RB5 and was used to investigate the decolorization of the dye. Therefore 12 plates were prepared.

**Table 2:** Four different types of plates were prepared for each of the three tested compounds (NAC-QDs, MPA-QDs, and CdCl_2_).

Plate number	Dye	Added compound	Fungus
1	No	Yes	No
2	RB5	Yes	No
3	No	Yes	Yes
4	RB5	Yes	Yes

Sterile LN-AS growth medium and the investigated compounds (NAC-QDs, MPA-QD, or CdCl_2_) with a final concentration of 0, 50, 100, 500, 1000, 2000, 5000, 7500, 10,000, 20,000 and 30,000 μg l^−1^ was achieved in each plate. In case of experiments containing RB5, the dye was dissolved in LN-AS growth medium so that the final concentration was 100 mg l^−1^ after adding all components of the assay (500 μl). In cases of setups that contained *P. velutina,* the experiments were started by adding 20 μl of the pre-growth mixture of the fungus, while adding 20 μl of deionized and sterile water to those without the fungus. The plates were covered by a lid and kept sterile, while incubating at 28 °C for 18 days. The plates were analyzed after 0, 6, 9, 14 and 18 days of incubation on a Tecan Infinite 200 plate reader (Tecan, Austria) at 597 nm (absorbance).

### 2.4 Kinetic calculations and statistical analysis

First order kinetic models were made for the decolorization reaction based on the initial absorbance A_*0*_ and at the respective incubation time A_*t*_ with k as the rate constant using the linearized form of a simple first order kinetic equation (Eq. (1).

$$-\ln\frac{A_{t}}{A_{0}}=kt$$

Eq. (1): Linearized first order decay function used for calculating the rate constants of decolorization reactions.

The formation of the compounds that were formed by the fungus in experiments without RB5 and absorbed light at 504 nm was calculated by a linearized first order growth curve Eq. (2). As the educt concentration was unknown, the maximum absorbance on day 18 was taken as the limiting value of the model.

$$\ln\left(1-\frac{A_{t}}{A_{\max}}\right)=kt$$

Eq. (2): Linearized first order growth function was used to calculate the rate constant for the formation of the unknown compounds that absorbed at 597 nm.

Half-lives (τ_1/2_) were calculated by ln (2)/k.

Statistical analysis for the determination of statistical significance of the data was performed in Sigma Plot 14.0.3 (Systat Software GmbH, Germany) using one- or two-way ANOVA with post-hoc Bonferroni test. A minimal level of p < 0.05 was regarded as significant.

Dose-response plots for the determination of EC_50_ values were fitted by four parametric logistic Hill's type curves (Eq. (3), also in Sigma Plot.

$$A=A_{\min}+\frac{A_{\max}-A_{\min}}{1+10^{\log\left({EC}_{50}-c\right)k_{Hill}}}$$

Eq. (3): Hill's type four parametric logistic curve with absorption (A), minimum absorption (A_min_), maximum absorption (A_max_), the EC_50_ value, the concentration of the metal salt (c), and the Hillslope (k_Hill_).

## 3 Results and discussion

### 3.1 Behavior of QDs in culture medium and on the reactive dyes RB5

NAC-QDs and MPA-QDs were analyzed in culture medium to investigate their stability and degradability during the incubation (Fig. S1 in the Supplementary Materials). A minor absorbance of NAC-QDs at 597 nm was found at the initial sampling point (0 days) for concentrations from 50 to 10,000 μg l^−1^, while MPA-QDs became visible only in a concentration range from 500 to 10,000 μg l^−1^. This indicates that MPA-QDs had a lower absorptivity than NAC-QDs. However, the absorbance of MPA-QDs was as low as that of the CdCl_2_ solution in the same concentration range. At concentrations >10,000 μg l^−1^ no absorbance could be measured, which indicates that the QDs formed aggregates and could not be detected. The absorbance of the QDs vanished with incubation time and could not be detected after 6 days. The absorption data remain independent of the QD concentration and developed into a stationary phase, which suggests that the culture medium rendered the particles unstable and only a fraction of the particles remained mobile, while the rest formed aggregates. For very high concentrations, the data indicated that the aggregation occurs instantaneously when the particles got into contact with the culture medium. A comparison of the coatings suggests that NAC-QDs were more stable than MPA-QDs. Overall, the absorbance of QDs vanished after 14 days entirely, independent from the coating. In case of CdCl_2_, the absorbance decreased to background level after 6 days.

Co-incubations of QDs with the reactive textile dye RB5, but without *P. velutina*, showed small and significant differences in the absorbance decays compared to all preparations with and without QDs or for CdCl_2_ (Fig. S2 in the Supplementary Materials). The degradation followed first order kinetic decays and reveal that RB5 oxidation was inhibited in the presence of NAC-QDs (τ_1/2_ = 196 - 136 d) and CdCl_2_ (τ_1/2_ = 146 - 55 d), while the oxidation was accelerated in the presence of MPA-QDs (τ_1/2_ = 127 - 34 d) in the concentration range from 50 to 20,000 μg l^−1^ compared to the half-lives in the absence of QDs or CdCl_2_ (τ_1/2_ = 197–68 d; Table S1 of the Supplementary materials). Since the concentration of MPA-QDs was lower than that of NAC-QDs (see Table 1), it can be expected that the accelerated decolorization of the dyes is related to the coating of the QDs. At 30,000 μg l^−1^, NAC-, MPA-QDs, and CdCl_2_ also accelerated the oxidation (τ_1/2_ = 73 - 66 d).

### 3.2 Impacts of QDs and CdCl_2_ on *P. velutina*

**Fig. 2:**
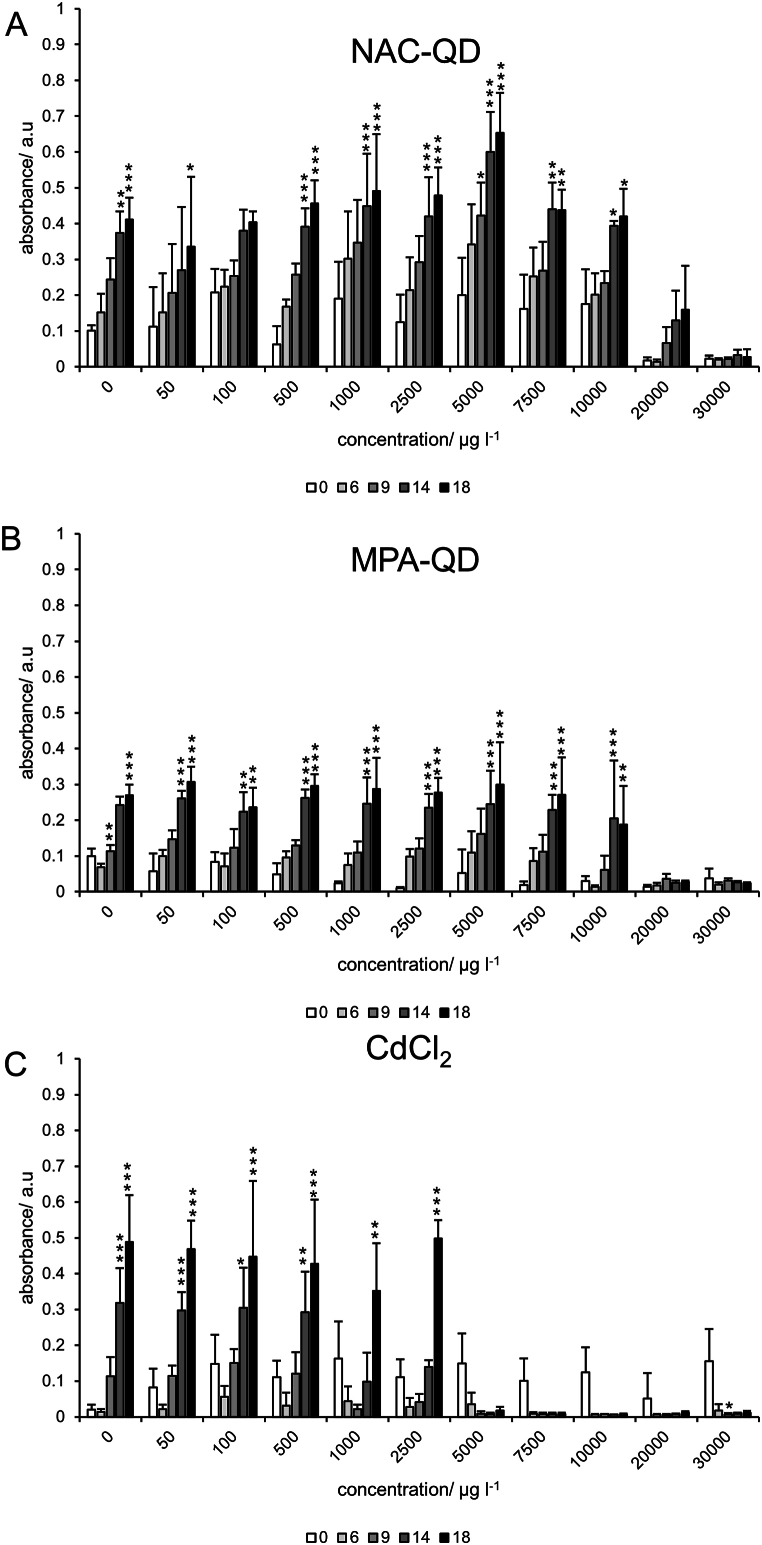
Absorbance of *P. velutina* incubated with (A) NAC-QDs, (B) MPA-QDs, and (C) CdCl_2_ without RB5, measured at 597 nm. The absorbance values represent the mean of quadruplicates, and the error bars the standard deviation. Significances of time and concentration were tested with a two-way ANOVA with post-hoc pairwise Bonferroni test. Significances are only shown for differences against the initial measurement (t = 0) with p < 0.05 with ∗, p < 0.01 with ∗∗, and p < 0.001 with ∗∗∗. Exact p values are given in the Supplementary Material Table. S4.

Earlier experiments on the decolorization efficacy of *P. velutina* on the textile dyes Reactive Orange 16 (RO16) and Remazol Brilliant Blue R (RBBR) showed interferences on the absorbance signals in the presence of fungi [16]. These interferences result in an increase of the absorbance signal, particularly when measuring the absorbance at the wavelength at which RBBR (492 nm) was analyzed. It is stressed that the interference can be observed at several wavelengths and originates only from compounds produced by the vital fungus in absence of any dyes [32]. The absorbance increase of the dye was dynamic and therefore the use of a single control at the start of the experiment (t = 0) was not sufficient to account for the absorbance increase. Therefore, a subtraction of the absorbance that is contributed by the fungus alone from the combined absorbance data was undertaken that allows to compensate the additional absorbance and obtain the decolorization performance only on the respective dye. This type of dynamic control experiment is shown in Fig. 2 for NAC-QDs, MPA-QDs and CdCl_2_ at the wavelength that was also used to analyze the decolorization of RB5 (597 nm). The data show a significant increase in absorbance for both types of QDs for concentrations <20,000 μg l^−1^ for day 14 and 18. In case of the NAC-QDs, non-significant absorbance increase was observed at 20,000 μg l^−1^. In the case of CdCl_2_, the concentration increase could be observed only for concentrations <5000 μg l^−1^, indicating that the Cd salt inhibited the production of the absorbing metabolites of the fungus.

### 3.3 Inhibition of the decolorization ability of *P. velutina* towards RB5 by QDs and CdCl_2_

**Fig. 3:**
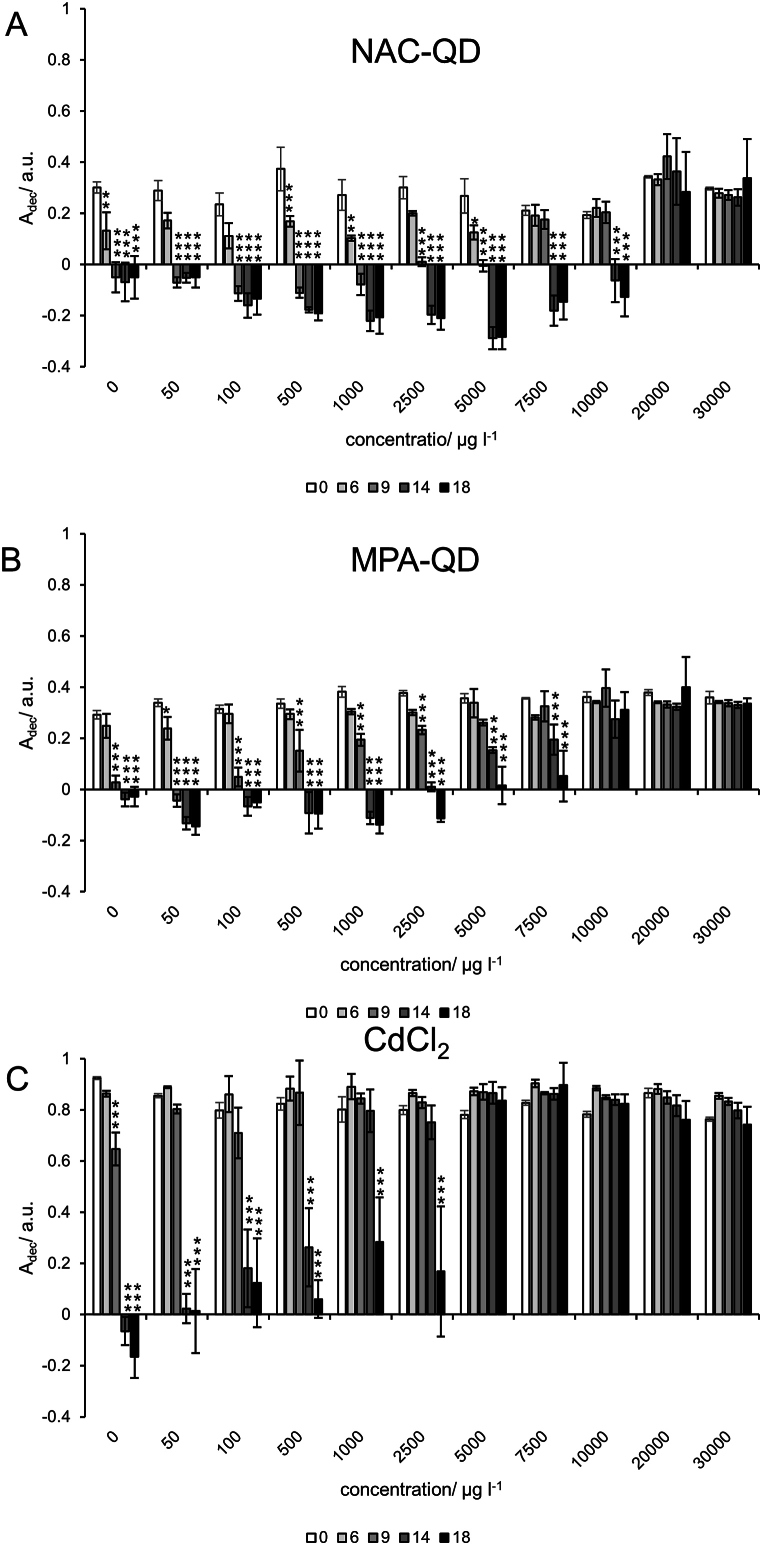
Decolorization of RB5 by *P. velutina* measured at 597 nm in presence of (A) NAC-QDs (B) MPA-QDs, and (C) CdCl_2_. The absorbance values (A_d__ec_) were obtained by subtracting the absorbance from experiments containing only *P. velutina* and without RB5 from absorbance data of *P. velutina* with RB5. A_d__ec_ values represent the mean of quadruplicates, and the error bars the standard deviation. Significances of time and concentrations was tested with a two-way ANOVA with post-hoc pairwise Bonferroni test. Significances are only shown for differences against the initial measurement (t = 0) with p < 0.05 with ∗, p < 0.01 with ∗∗, and p < 0.001 with ∗∗∗. Exact p values are given in Table S5 of the Supplementary Materials.

Changes in absorption of the textile dye RB5 with *P. velutina* show different trends in the co-presence and absence of NAC-, MPA-QDs, and CdCl_2_ (Fig. S3 in the Supplemental Materials). Experiments with NAC-QDs show a minimum at day 6–9 in the absorbance transients in the concentration range 0–5000 μg l^−1^. In case of MPA-QDs, the absorbance declined for concentrations 0–2500 μg l^−1^, as in case of CdCl_2_. Additionally, CdCl_2_ shows a distinct lower absorbance decrease with increasing concentration. These different observations suggest that the decolorization of RB5 and the increase of absorbance, by the metabolites produced by *P. velutina* (cf. Fig. 2), occurred simultaneously and that the NAC-, MPA-QDs, and CdCl_2_ inhibited the decolorization to different extents. The data obtained from the experiment with *P. velutina* and without RB5 allows to assess the extent of the dynamic absorbance increase by the metabolites and a subtraction of the increase from the decolorization data (Fig. 3). Fig. 3 shows that the subtraction of the decolorization data from the absorption data, which indicates metabolite production (Fig. 2), can also result in negative values. In these cases, the absorption increase was higher than the extent of decolorization, which is most probably caused by inhibitory effects of the dyes that in turn affects the vitality of *P. velutina*.

The subtracted absorbance data shows that *P. velutina* was able to decolorize RB5 co-exposed with NAC-QDs in the concentration range from 0 to 5000 μg l^−1^ entirely after 6 days and between 5000 and 10000 μg l^−1^ after 9 days, while no decolorization took place at NAC-QD concentrations >10000 μg l^−1^. In the presence of MPA-QDs, RB5 was decolorized entirely after 6 days only at 50 μg l^−1^, while in a range from 100–2500 μg l^−1^ the decolorization was achieved after 9 days and at 5000–7500 μg l^−1^ after 14 days. Concentration >7500 μg l^−1^ inhibited the decolorization of RB5 completely. The strongest inhibition was found for CdCl_2_ that shows that an entire decolorization was possible only for CdCl_2_ at a concentration of 50 μg l^−1^. At 100 and 500 μg l^−1^ a significant decolorization could be observed after 14 days, and at 1000 and 2500 μg l^−1^. At concentrations >2500 μg l^−1^ no decolorization were observed within 18 days.

### 3.4 Dose-response curves calculated from decolorization assays

The extend of inhibition of decolorization of RB5 by *P. velutina* by NAC-, MPA-QDs and CdCl_2_ was assessed by the EC_50_ values obtained from dose-response calculations based on 4 parametric logistic Hill's type models (Eq. (3). These calculations were made for each measured point in time for the absorbance increase and the subtracted decolorization data (Supplementary Material Fig. S4 and S5, respectively). The coefficients of determinations and the p-value of the EC_50_ value show that the dose-response curves returned good fits (R^2^ > 0.47 and p < 0.05) of the absorbance increase for incubation of 9, 14, and 18 days for NAC- and MPA-QDs, as well as, for 9 and 14 days for CdCl_2_ (Supplementary Material Table S6). The decolorization data shows better fits than the growth curves for 9–18 days (Supplementary Material Tab. S7) and the results of the best fits (regarding R^2^ and the p-values) are presented in Table 3. Generally, these results show that the EC_50_ for the decolorization (dec) was a factor of 2.4, 2.3, and 1.9 times lower for NAC-QDs, MPA-QDs, and CdCl_2_, respectively, which indicates that inhibition of decolorization was more sensitive than the formation of the metabolites. This suggests that extracellular enzymes, which are excreted by the fungus, were less abundant and/or deactivated and were not able to decolorize RB5 anymore, while the fungus was still producing metabolites, which can be regarded as a hallmark of the vitality of the fungus. Regarding the differences in EC_50_ values for inhibiting the decolorization of RB5, NAC-QDs had the lowest impact with 7262 μg l^−1^ (161 nM QD), while the EC_50_ of MPA-QDs was almost half (4694 μg μl^−1^; 5 nM QD). For the absorption increase, EC_50_ values of 17,575 μg l^−1^ (391 nM) and 10,628 μg l^−1^ (10 nM) were found for NAC-QDs and MPA-QDs, respectively. In case of the NAC-QDs (2.9 nm), the EC_50_ values was higher than those reported on yeast, *Saccharomyces cerevisiae,* for NAC-QD (CdTe) with an IC_50_ on growth inhibition of 17 and 81 nM for the diameters 5.8 nm and 4.1 nm, respectively [33], which correlates to the size difference (diameters: 5.8, 4.1, and 2.9 nm compared to IC_50_: 17, 81, and 391 nM). However, the IC_50_ of 304 nM (diameter ca. 4 nm) for octylamine polyacrylic acid (OPA) coated CdSe/ZnS QDs [34], is much higher than the values found for MPA-QDs (CdTe/ZnS) with and EC_50_ of 10 nM, which may be also associated with the size of the QDs (diameters: ⁓4 and 8.3 nm resulted in IC_50_ values of 304 and 10 nM, respectively). The comparison with literature data shows that the values are very most likely associated with the size of QDs than with the coating or the fungal species.

**Table 3:** Results from the best fits of the EC_50_ curves (4 parametric logistic curves; Hill's type) of absorption data from the decolorization assay (dec) and the experiment without RB5 (increase).

	Type	time/d	EC_50_/μg l^−1^ (p-value)	min/μg l^−1^ (p-value)	max/μg l^−1^ (p-value)	Hillslope (p-value)	R^2^
dec	NAC-QD	9	7262.13 (<0.0001)	−0.074 (<0.0001)	0.3484 (<0.0001)	3.2086 (0.0013)	0.8742
	MPA-QD	14	4694.24 (<0.0001)	−0.0894 (<0.0001)	0.3387 (<0.0001)	2.1051 (0.0002)	0.9036
	CdCl_2_	14	583.18 (<0.0001)	0.0463 (0.1022)	0.8232 (<0.0001)	6.1623 (0.0382)	0.9362
increase	NAC-QD	18	17575.40 (<0.0001)	−0.0029 (0.982)	0.4578 (<0.0001)	−4.8815 (0.2832)	0.5392
	MPA-QD	18	10628.20 (<0.0001)	0.0249 (0.3743)	0.282 (<0.0001)	−9.0361 (0.5218)	0.6425
	CdCl_2_	14	1119.11 (0.0019)	0.0005 (0.9854)	0.3151 (<0.0001)	−1.473 (0.0218)	0.7645

CdCl_2_ had the strongest impact with an EC_50_ of 583 μg l^−1^ (3.2 μM), which was an order of magnitude lower than for both types of QDs. However, QDs cannot be readily compared to CdCl_2_, as the nanoparticulate QDs do not consist entirely of Cd, but also of Te. Therefore, an estimation of the Cd concentration was made by using geometric parameters of the QDs and the bulk density of CdTe (Table 1). This calculation results in a Cd content of 47 m%, which means that the EC_50_ of the Cd in NAC-QDs would correspond to 3401 μg l^−1^ and for MPA-QDs to 2199 μg l^−1^. These EC_50_ values were still 3.8–5.8 times larger than that of dissolved CdCl_2_, which suggests that the QDs were not entirely dissolved or decomposed during the experiment, which may also be associated with a precipitation of the QDs in the culture medium. A direct measurement in the growth medium was not possible, therefore an experiment was made in a comparable buffer consisting of citrate and phosphate buffers at pH 4.5. In this experiment, a ready dissolution of the QDs was observed (Fig. S 6 of the Supplementary Materials), which indicates that both types of QDs are unstable after a short time at low pH and that dissolution is one of the involved mechanisms. However, a growth medium consists also of organic components (polysaccharides, proteins, etc.) that could also stabilize the nanoparticles and prevent temporarily the dissolution process. Therefore, a possible mechanism could be that the particles were stabilized temporarily by the organic matter in the growth medium and slowly dissolved, which could have led to lower EC_50_ concentration for the QDs.

The simplest possible mechanism for inhibiting the decolorization is to assume that it is solely caused by Cd ions that originate from the dissolution of the QD's particle core. Under such environmental conditions, the results of the approximated EC_50_ for the Cd content in NAC- and MPA-QDs suggests that 17 % of NAC-QDs and 27 % of MPA-QDs were dissolved. This different solubility rates can be explained by the passivation properties of the nanoparticle coating, their steric stabilization effect, and different grafting densities of the used organic ligands. Moreover, the mechanisms that inhibit decolorization can be more complex for engineered nanoparticles, such as QDs. A possible toxic mechanism could be their bioavailability and the penetration of the fungi cell membranes, as also reported for eukaryotic cells [31]. Considering the Cd content and adjusting the EC_50_ values of the absorbance increase to the Cd concentration, resulted in 8231 μg l^−1^ and 4978 μg l^−1^ for NAC- and MPA-QDs, respectively, which were 7.4 and 4.5 times higher than the EC_50_ value of CdCl_2_. The observed inhibition of the absorbance increase corresponds therefore to ∼14 % of dissolved Cd from NAC-QDs and 23 % from MPA-QDs in relation to the EC_50_ of CdCl_2_ (1119 μg l^−1^, 6.1 μM). The comparison of the dissolved Cd fraction of NAC- and MPA-QDs between decolorization and absorbance increase suggests that the QDs were less inhibitory towards the formation of the metabolites (absorbance increase) than towards decolorization in presence of CdCl_2_. Mei et al. [34] also investigated the inhibitory effect of Cd on the growth of yeast *S. cerevisiae* and found an IC_50_ value of 3.3 μM, which compares well with the values found for decolorization (3.2 μM) and absorption increase (6.1 μM) in this case of the growth of the white rot fungus *P. velutina*. Assuming that the absorption increase (metabolite production) reflects the vitality of the fungus, it is suggested that *P. velutina* is more resistant towards Cd than *S. cerevisiae*.

## 4 Conclusion

Basidiomycetous fungi such as *P. velutina* are promising candidates for bioremediation and wastewater treatment to decompose persistent organic pollutants, such as synthetic dyes. *P. velutina was* shown to be able to decompose and decolorize aromatic synthetic dye compounds when they are exposed to co-pollutants such as heavy metals and differently coated hydrophilic quantum dots. However, the co-presence of nanoparticles led to an inhibition of the decolorization, which was 3.8–5.8 times lower, than when they were exposed to dissolved Cd. Between the QDs, it was not possible to assess whether the coating or the particle size of the NAC-QDs and MPA-QDs resulted in a greater impact on the nano-specific toxic effects. The EC_50_ values also showed that *P. velutina* was more resilient than other investigated fungal species in literature towards soluble Cd pollution, which could make it useful to depollute wastewater. Future studies should systematically investigate the size dependence and different coatings of nanoparticles on *P. velutina* or other fungal species to elucidate the determining factor on nanotoxicity. In addition, speciation of metabolites and environmental risk assessment, especially of possible degradation products, should be carried out before translating the results to pilot scale applications in order to assess the performance and unknown risks of bioremediation applications for wastewater and contaminated soils.

## CRediT authorship contribution statement

**Florian Part:** Writing – review & editing, Writing – original draft, Investigation, Data curation, Conceptualization. **Christian Zafiu:** Writing – review & editing, Writing – original draft, Visualization, Methodology, Formal analysis, Data curation, Conceptualization. **Eva-Kathrin Ehmoser:** Writing – review & editing, Conceptualization. **Mika A. Kähkönen:** Writing – review & editing, Writing – original draft, Supervision, Resources, Investigation, Conceptualization.

## Data availability

The authors declare that the data will be made available on request.

## Declaration of generative AI in scientific writing

The authors declare that no generative artificial intelligence (AI) and AI-assisted technologies were used in the writing process.

## Declaration of competing interest

The authors declare the following financial interests/personal relationships which may be considered as potential competing interests:Mika Kaehkoenen reports financial support was provided by Department of Microbiology (Biocenter 1, Viikinkaari 9), Faculty of Agriculture and Forestry, 10.13039/100007797University of Helsinki, Finland. Christian Zafiu reports article publishing charges was provided by University of Natural Resources and Life Sciences, Vienna, Department of Water-Atmosphere-Environment, Institute of Waste Management and Circularity. If there are other authors, they declare that they have no known competing financial interests or personal relationships that could have appeared to influence the work reported in this paper.
