# Supplementary material for: Inhibitory effects of cadmium and hydrophilic cadmium telluride quantum dots on the white rot fungus Phanerochaete velutina
Source: Heliyon. 2024 Dec 12;11(1):e41190. doi: 10.1016/j.heliyon.2024.e41190 (PMC11730851; doi:10.1016/j.heliyon.2024.e41190)
Supplement: Multimedia component 1 [file mmc1.docx]

**Supplementary materials: Inhibitory effects of cadmium and hydrophilic cadmium telluride quantum dots on the white rot fungus *Phanerochaete velutina***


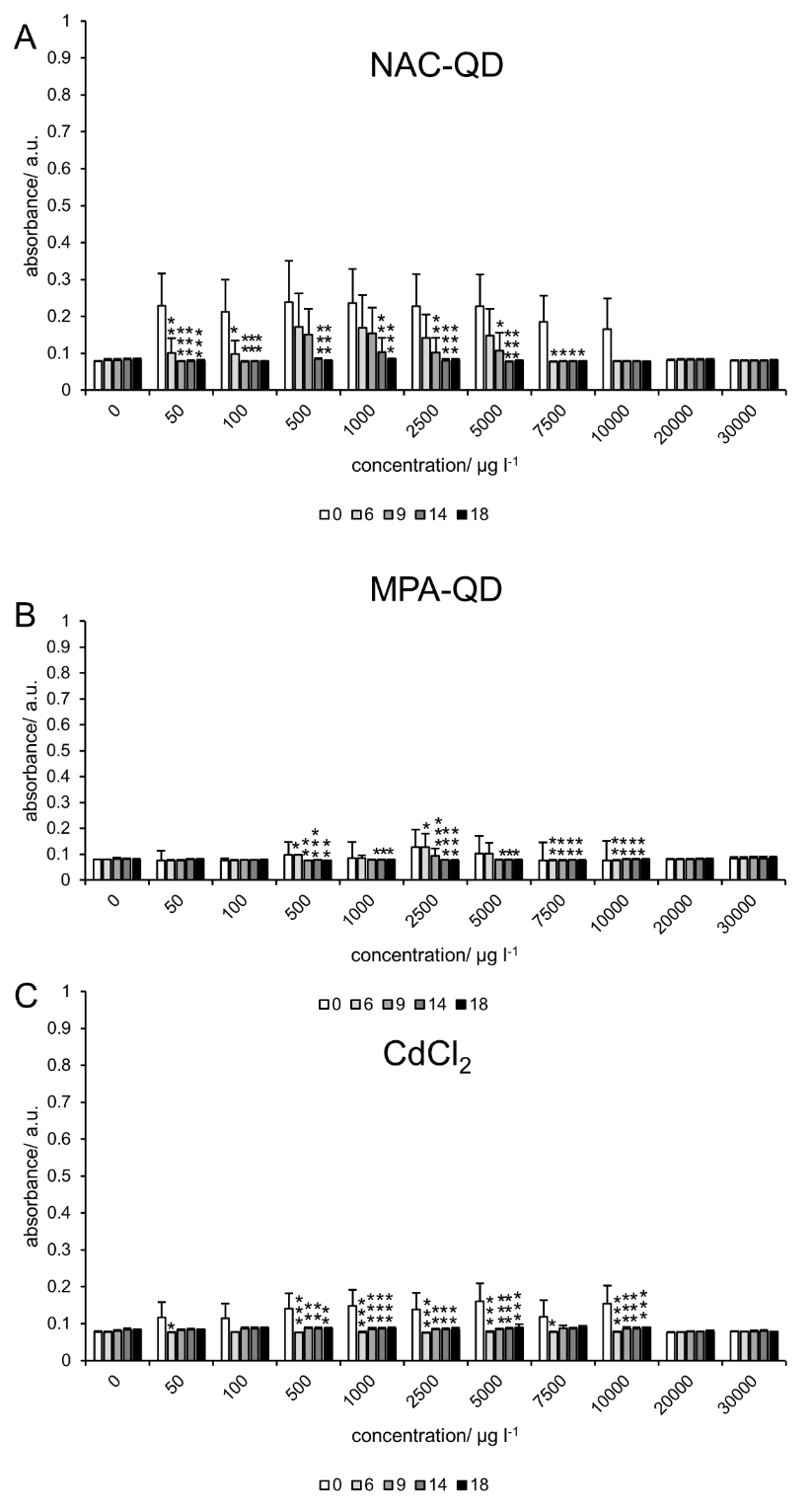


Fig.S 1: Absorbance of (A) NAC-QDs (B) MPA-QDs, and (C) CdCl_2_ in pure culture medium measured at 597 nm. The absorbance values represent the mean of quadruplicates, and the error bars the standard deviation. Significance was tested with a two-way ANOVA for time and concentration with post-hoc pair wise Bonferroni test. Significances are only shown for differences against the initial time point t=0 with p < 0.05 with *, p < 0.01 with **, and p < 0.001 with ***. The exact p values are given in Tab. S 2.


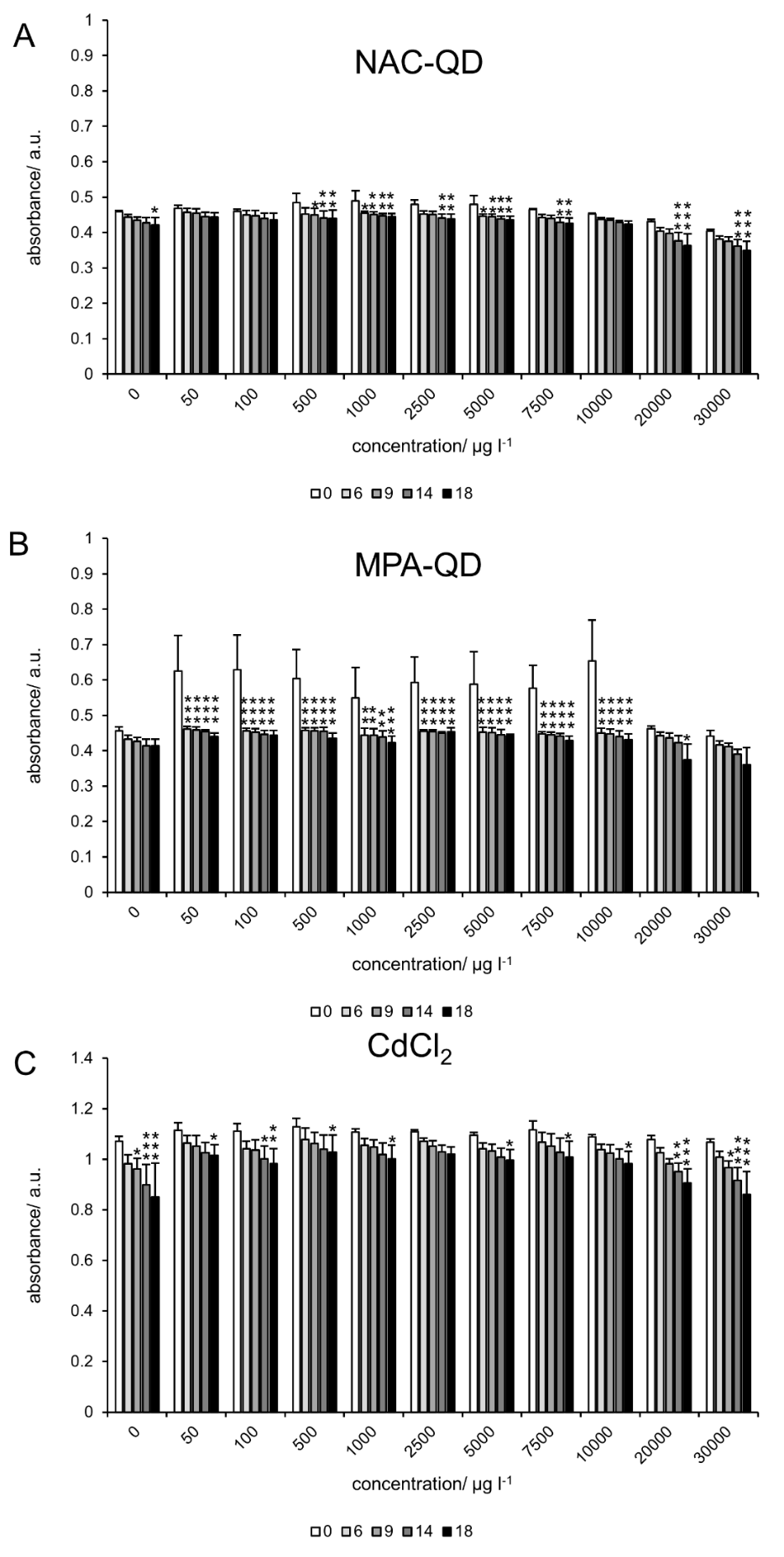


Fig.S 2: Absorbance of (A) NAC-QDs (B) MPA-QDs, and (C) CdCl_2_ in pure culture medium containing RB5 and measured at 597 nm. The absorbance values represent the mean of quadruplicates, and the error bars the standard deviation. Significance was tested with a two-way ANOVA for time and concentration with post-hoc pair wise Bonferroni test. Significances are only shown for differences against the initial time point t=0 with p < 0.05 with *, p < 0.01 with **, and p < 0.001 with ***. The exact p values are given in Tab. S 3.


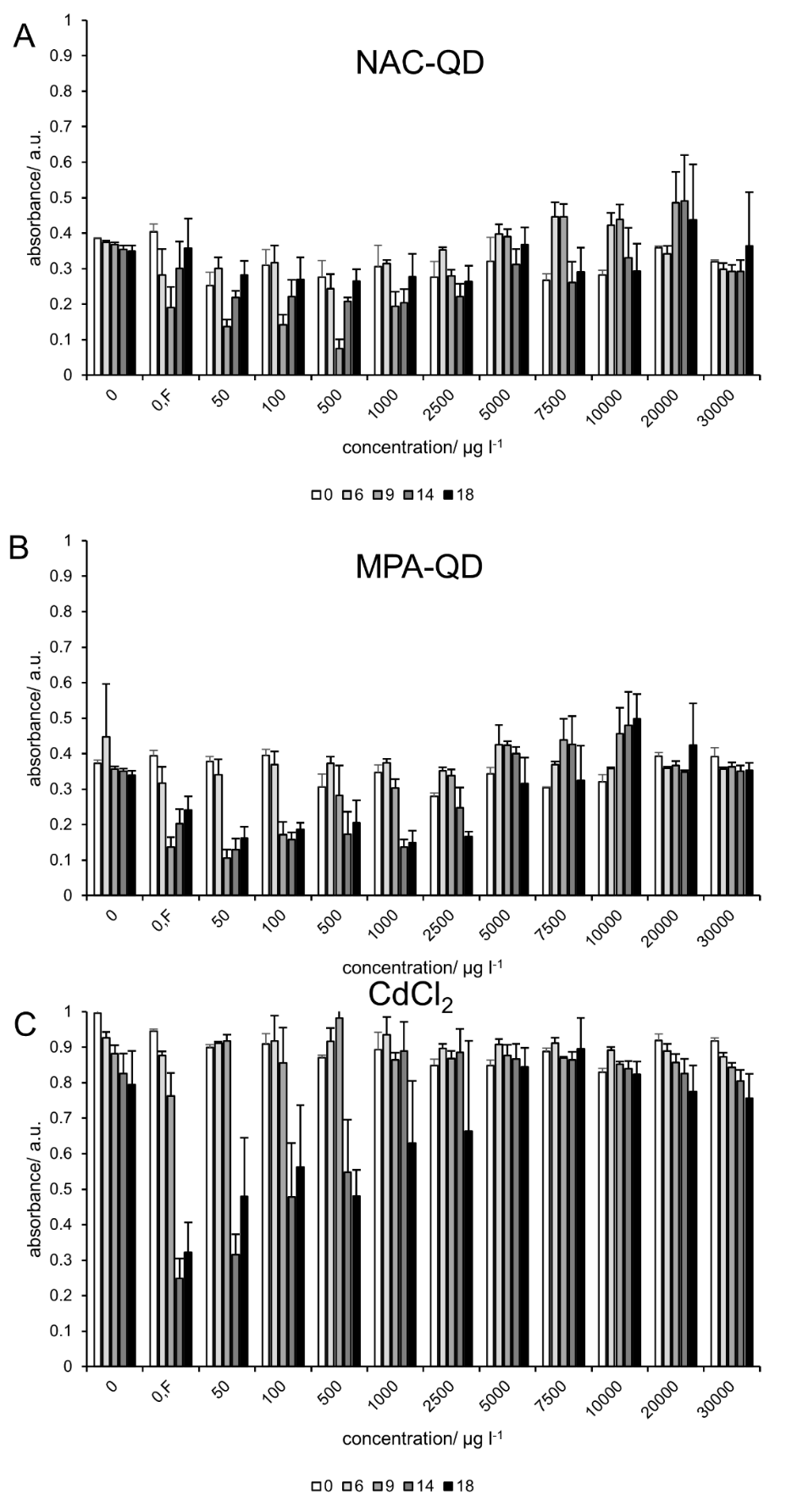


Fig.S 3: Absorbance of *P. velutina* incubated with (A) NAC-QDs (B) MPA-QDs, and (C) CdCl_2_ in the presence of RB5, measured at 597 nm. The absorbance values represent the mean of quadruplicates, and the error bars the standard deviation. The sample “0” contained only RB5 in the growth medium, while “0,F” contained RB5 and *P. velutina*. Significance was tested with a two-way ANOVA for time and concentration with post-hoc pair wise Bonferroni test. Significances are only shown for differences against the initial time point t=0 with p < 0.05 with *, p < 0.01 with **, and p < 0.001 with ***.


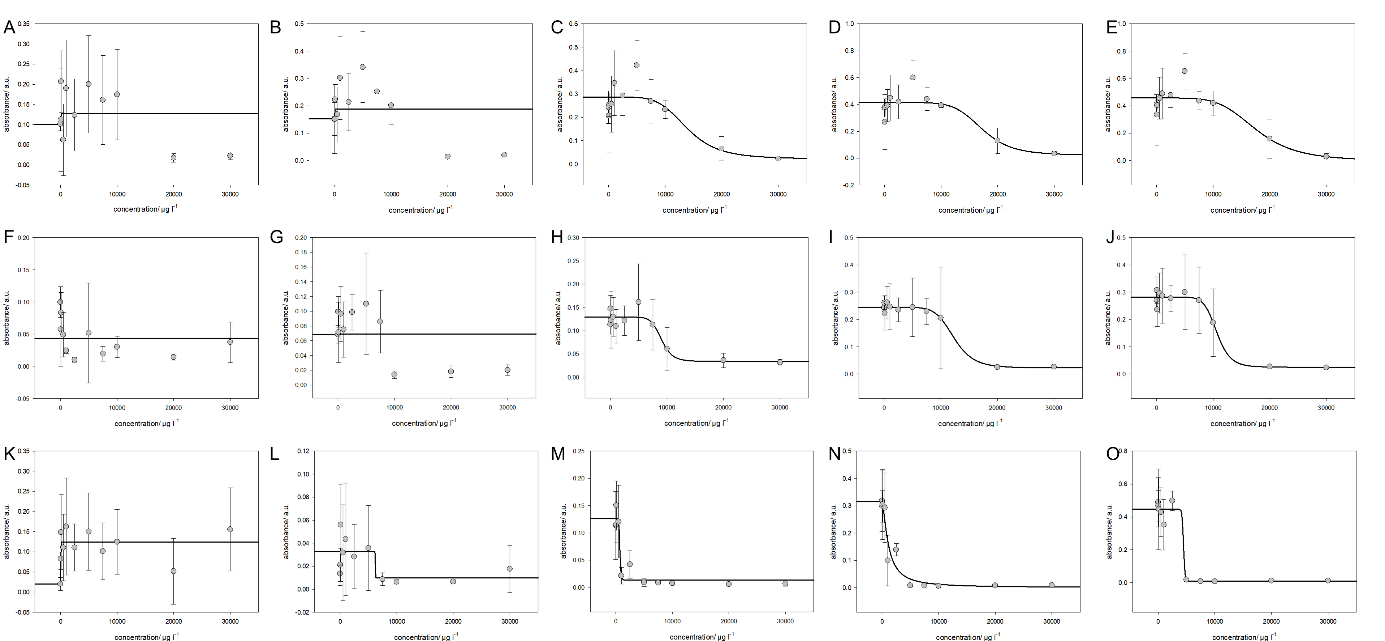


Fig.S 4: 4 parametric logistic curves (Hill’s type) of the absorbance data from experiments with of *P. velutina* without RB5. The upper panel shows NAC-QD after 0 (A), 6 (B), 9 (C), 14 (D), and 18 (E) days. The middle panel shows MPA-QD after 0 (F), 6 (G), 9 (H), 14 (I), and 18 (J) days and the bottom panel shows CdCl_2_ after 0 (K), 6 (L), 9 (M), 14 (N), and 18 (O) days. The error bars represent the standard deviation of quadruplicates.


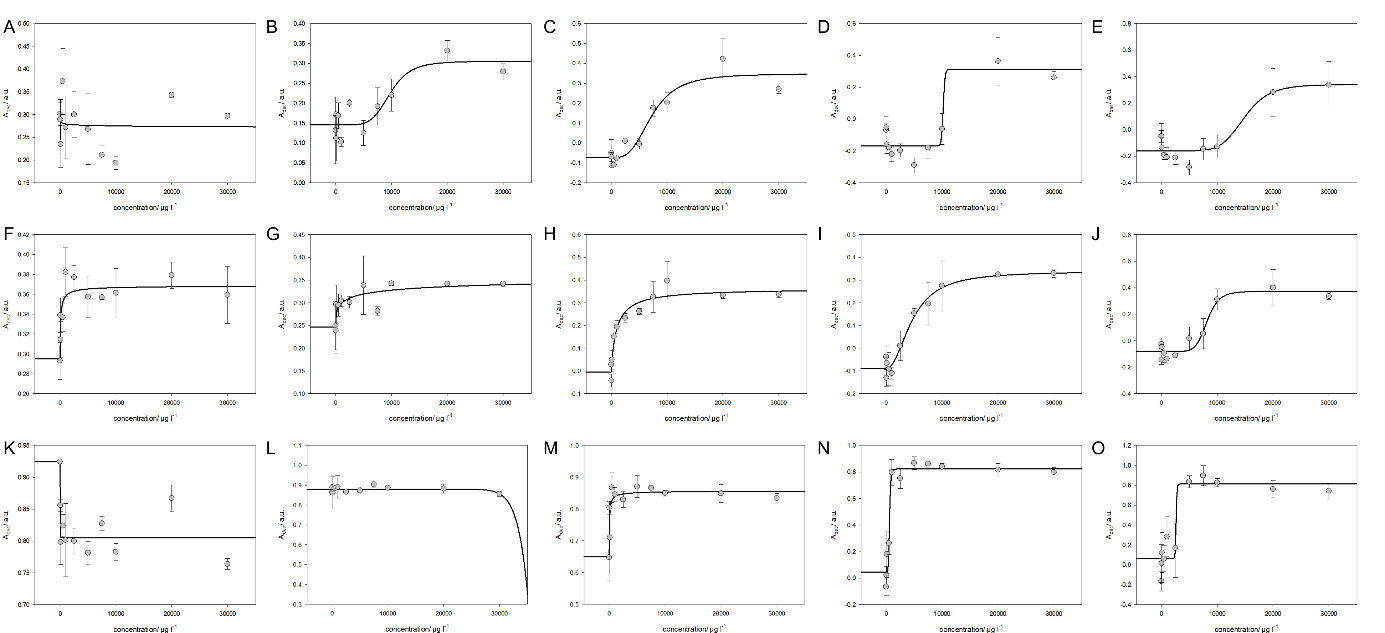


Fig.S 5: 4 parametric logistic curves (Hill’s type) of the absorbance data from experiments with of *P. velutina* with RB5 after subtraction of the absorbance increase data form experiments without RB5. The upper panel shows NAC-QD after 0 (A), 6 (B), 9 (C), 14 (D), and 18 (E) days. The middle panel shows MPA-QD after 0 (F), 6 (G), 9 (H), 14 (I), and 18 (J) days and the bottom panel shows CdCl_2_ after 0 (K), 6 (L), 9 (M), 14 (N), and 18 (O) days. The error bars represent the standard deviation of quadruplicates.


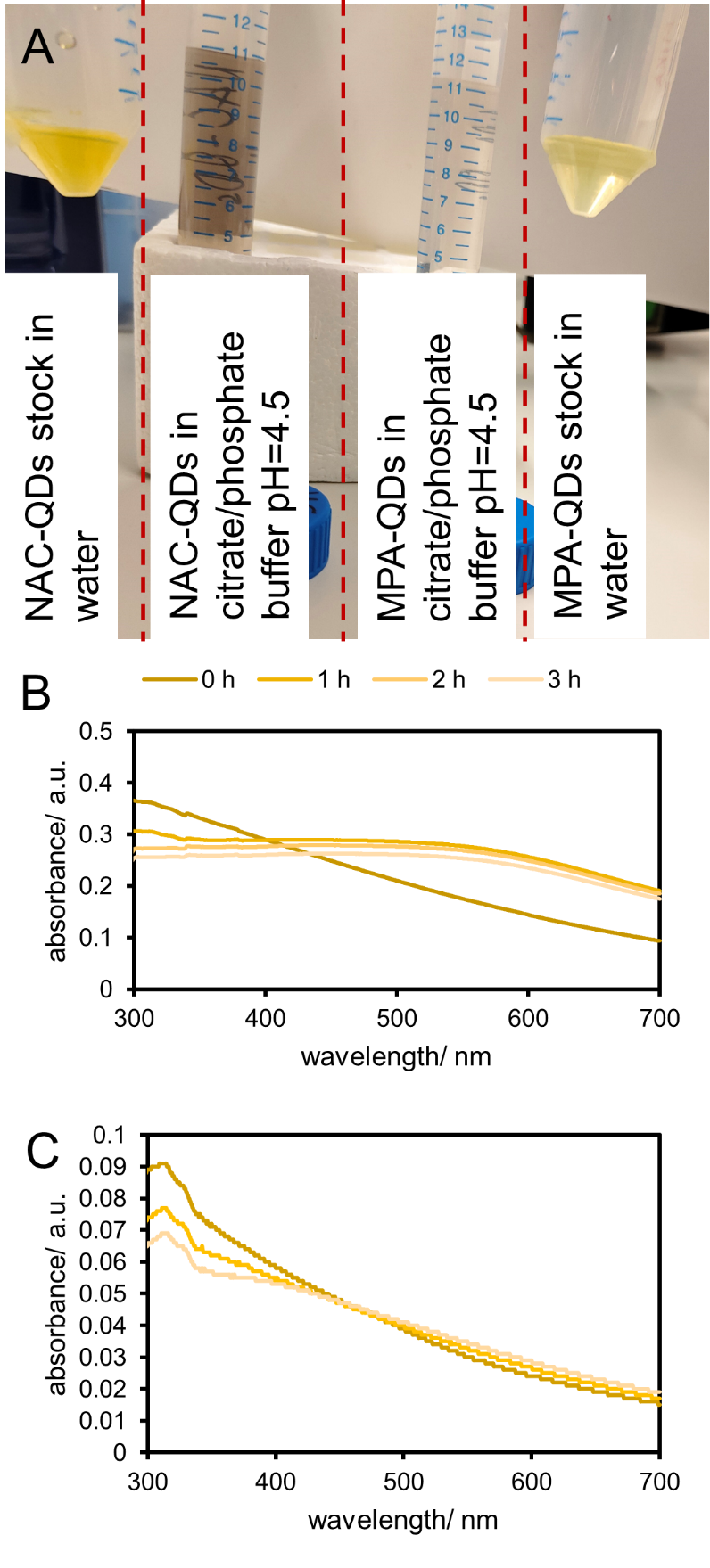


Fig.S 6: A) Photographs of NAC-QDs in water (left), NAC-QDs in citrate/phosphate buffer pH=4.5 (mid left), MPA-QDs in citrate/phosphate buffer pH=4.5 (mid right), and MPA-QDs in water (right). B) UV-VIS spectra of MPA-QDs and C) NAC-QDs in citrate/phosphate buffer pH=4.5 after 0, 1, 2, and 3 h.

Tab. S 1: Results from first order kinetic decay calculation of the oxidative degradation of RB5 in the presence of NAC-QDs, MPA-QDs, and CdCl_2_ in pure culture medium without *P. velutina*, with the rate constant (k), the coefficient of determination (R²) and the half-life (τ_1/2_).

|  | k/ d^-1^ | | | R² | | | τ_1/2_/ d | | |
| --- | --- | --- | --- | --- | --- | --- | --- | --- | --- |
| C/ µg l^-1^ | NAC-QDs | MPA-QDs | CdCl_2_ | NAC-QDs | MPA-QDs | CdCl_2_ | NAC-QDs | MPA-QDs | CdCl_2_ |
| 0 | 0.0054 | 0.0035 | 0.0102 | 0.9825 | 0.7565 | 0.9943 | 129.3 | 196.7 | 67.7 |
| 50 | 0.0047 | 0.0055 | 0.0126 | 0.9837 | 0.9271 | 0.9955 | 148.5 | 126.5 | 55.0 |
| 100 | 0.0032 | 0.0171 | 0.0051 | 0.9716 | 0.6794 | 0.9666 | 214.7 | 40.6 | 134.8 |
| 500 | 0.0028 | 0.0171 | 0.0066 | 0.9907 | 0.6338 | 0.9566 | 243.6 | 40.4 | 105.6 |
| 1000 | 0.0051 | 0.0157 | 0.0051 | 0.8334 | 0.6868 | 0.9632 | 135.8 | 44.3 | 135.7 |
| 2500 | 0.0049 | 0.0127 | 0.0055 | 0.7614 | 0.7281 | 0.9775 | 140.5 | 54.5 | 127.0 |
| 5000 | 0.0048 | 0.0131 | 0.0047 | 0.8775 | 0.5760 | 0.9767 | 143.4 | 53.1 | 146.3 |
| 7500 | 0.0049 | 0.0139 | 0.0051 | 0.7995 | 0.6352 | 0.9591 | 142.7 | 49.8 | 135.3 |
| 10000 | 0.0047 | 0.0145 | 0.0056 | 0.9337 | 0.6875 | 0.9846 | 146.1 | 47.8 | 124.1 |
| 20000 | 0.0035 | 0.0203 | 0.0056 | 0.9668 | 0.6550 | 0.9768 | 195.9 | 34.2 | 124.2 |
| 30000 | 0.0095 | 0.0105 | 0.0096 | 0.9946 | 0.8421 | 0.9908 | 73.2 | 65.8 | 72.4 |

Tab. S 2: Results from two-way ANOVA on quadruplicate absorbance data (597 nm) of NAC-QDs, MPA-QDs, and CdCl_2_ in pure culture medium using timepoints and concentration. For pairwise t-test the Bonferroni post-hoc test was used. Only significant comparisons with p<0.05 are shown.

| Type | C/ µg l^-1^ | Comp. | Diff. of means | t | p |
| --- | --- | --- | --- | --- | --- |
| NAC-QD | 50 | 0 vs. 6 | 0.127 | 3.558 | 0.005 |
| NAC-QD | 50 | 0 vs. 9 | 0.15 | 4.21 | <0.001 |
| NAC-QD | 50 | 0 vs. 14 | 0.151 | 4.215 | <0.001 |
| NAC-QD | 50 | 0 vs. 18 | 0.148 | 4.153 | <0.001 |
| NAC-QD | 100 | 0 vs. 6 | 0.114 | 3.184 | 0.017 |
| NAC-QD | 100 | 0 vs. 9 | 0.134 | 3.758 | 0.002 |
| NAC-QD | 100 | 0 vs. 14 | 0.134 | 3.745 | 0.002 |
| NAC-QD | 100 | 0 vs. 18 | 0.133 | 3.735 | 0.003 |
| NAC-QD | 500 | 0 vs. 14 | 0.154 | 4.302 | <0.001 |
| NAC-QD | 500 | 0 vs. 18 | 0.159 | 4.458 | <0.001 |
| NAC-QD | 1000 | 0 vs. 14 | 0.133 | 3.723 | 0.003 |
| NAC-QD | 1000 | 0 vs. 18 | 0.152 | 4.247 | <0.001 |
| NAC-QD | 2500 | 0 vs. 9 | 0.125 | 3.506 | 0.006 |
| NAC-QD | 2500 | 0 vs. 14 | 0.147 | 4.119 | <0.001 |
| NAC-QD | 2500 | 0 vs. 18 | 0.146 | 4.085 | <0.001 |
| NAC-QD | 5000 | 0 vs. 9 | 0.12 | 3.361 | 0.01 |
| NAC-QD | 5000 | 0 vs. 14 | 0.15 | 4.19 | <0.001 |
| NAC-QD | 5000 | 0 vs. 18 | 0.147 | 4.11 | <0.001 |
| NAC-QD | 7500 | 0 vs. 6 | 0.108 | 3.014 | 0.03 |
| NAC-QD | 7500 | 0 vs. 9 | 0.107 | 2.986 | 0.033 |
| NAC-QD | 7500 | 0 vs. 14 | 0.107 | 2.992 | 0.032 |
| NAC-QD | 7500 | 0 vs. 18 | 0.106 | 2.981 | 0.033 |
| MPA-QD | 500 | 0 vs. 6 | 0.0606 | 2.955 | 0.036 |
| MPA-QD | 500 | 0 vs. 9 | 0.0818 | 3.99 | <0.001 |
| MPA-QD | 500 | 0 vs. 14 | 0.0807 | 3.935 | 0.001 |
| MPA-QD | 500 | 0 vs. 18 | 0.0819 | 3.993 | <0.001 |
| MPA-QD | 1000 | 0 vs. 9 | 0.0603 | 2.941 | 0.037 |
| MPA-QD | 1000 | 0 vs. 14 | 0.0602 | 2.935 | 0.038 |
| MPA-QD | 1000 | 0 vs. 18 | 0.0602 | 2.936 | 0.038 |
| MPA-QD | 2500 | 0 vs. 6 | 0.0588 | 2.868 | 0.047 |
| MPA-QD | 2500 | 0 vs. 9 | 0.0923 | 4.503 | <0.001 |
| MPA-QD | 2500 | 0 vs. 14 | 0.109 | 5.301 | <0.001 |
| MPA-QD | 2500 | 0 vs. 18 | 0.109 | 5.327 | <0.001 |
| MPA-QD | 5000 | 0 vs. 9 | 0.067 | 3.268 | 0.013 |
| MPA-QD | 5000 | 0 vs. 14 | 0.0671 | 3.272 | 0.013 |
| MPA-QD | 5000 | 0 vs. 18 | 0.0666 | 3.247 | 0.014 |
| MPA-QD | 7500 | 0 vs. 6 | 0.073 | 3.558 | 0.005 |
| MPA-QD | 7500 | 0 vs. 9 | 0.0729 | 3.552 | 0.005 |
| MPA-QD | 7500 | 0 vs. 14 | 0.0724 | 3.529 | 0.005 |
| MPA-QD | 7500 | 0 vs. 18 | 0.0726 | 3.541 | 0.005 |
| MPA-QD | 10000 | 0 vs. 6 | 0.0723 | 3.524 | 0.006 |
| MPA-QD | 10000 | 0 vs. 9 | 0.0699 | 3.411 | 0.008 |
| MPA-QD | 10000 | 0 vs. 14 | 0.0692 | 3.374 | 0.009 |
| MPA-QD | 10000 | 0 vs. 18 | 0.0696 | 3.391 | 0.009 |
| CdCl_2_ | 50 | 0 vs. 6 | 0.041 | 2.939 | 0.038 |
| CdCl_2_ | 50 | 0 vs. 6 | 0.0651 | 4.669 | <0.001 |
| CdCl_2_ | 50 | 0 vs. 9 | 0.0534 | 3.832 | 0.002 |
| CdCl_2_ | 50 | 0 vs. 14 | 0.0546 | 3.913 | 0.001 |
| CdCl_2_ | 50 | 0 vs. 18 | 0.0537 | 3.85 | 0.002 |
| CdCl_2_ | 500 | 0 vs. 6 | 0.0651 | 4.669 | <0.001 |
| CdCl_2_ | 500 | 0 vs. 9 | 0.0534 | 3.832 | 0.002 |
| CdCl_2_ | 500 | 0 vs. 14 | 0.0546 | 3.913 | 0.001 |
| CdCl_2_ | 500 | 0 vs. 18 | 0.0537 | 3.85 | 0.002 |
| CdCl_2_ | 1000 | 0 vs. 6 | 0.0717 | 5.143 | <0.001 |
| CdCl_2_ | 1000 | 0 vs. 9 | 0.064 | 4.594 | <0.001 |
| CdCl_2_ | 1000 | 0 vs. 14 | 0.0626 | 4.49 | <0.001 |
| CdCl_2_ | 1000 | 0 vs. 18 | 0.0616 | 4.416 | <0.001 |
| CdCl_2_ | 2500 | 0 vs. 6 | 0.0631 | 4.528 | <0.001 |
| CdCl_2_ | 2500 | 0 vs. 9 | 0.0535 | 3.841 | 0.002 |
| CdCl_2_ | 2500 | 0 vs. 14 | 0.0542 | 3.884 | 0.001 |
| CdCl_2_ | 2500 | 0 vs. 18 | 0.0551 | 3.952 | 0.001 |
| CdCl_2_ | 5000 | 0 vs. 6 | 0.0827 | 5.93 | <0.001 |
| CdCl_2_ | 5000 | 0 vs. 9 | 0.0754 | 5.408 | <0.001 |
| CdCl_2_ | 5000 | 0 vs. 14 | 0.0742 | 5.32 | <0.001 |
| CdCl_2_ | 5000 | 0 vs. 18 | 0.0708 | 5.082 | <0.001 |
| CdCl_2_ | 7500 | 0 vs. 6 | 0.0408 | 2.926 | 0.039 |
| CdCl_2_ | 10000 | 0 vs. 6 | 0.0773 | 5.544 | <0.001 |
| CdCl_2_ | 10000 | 0 vs. 9 | 0.0682 | 4.89 | <0.001 |
| CdCl_2_ | 10000 | 0 vs. 14 | 0.0693 | 4.972 | <0.001 |
| CdCl_2_ | 10000 | 0 vs. 18 | 0.0656 | 4.703 | <0.001 |

Tab. S 3: Results from two-way ANOVA on quadruplicate absorbance data (597 nm) of NAC-QDs, MPA-QDs, and CdCl_2_ in pure culture medium containing RB5 using timepoints and concentration. For pairwise t-test the Bonferroni post-hoc test was used. Only significant comparisons with p<0.05 are shown.

| Type | C/ µg l^-1^ | Comp. | Diff. of means | t | p |
| --- | --- | --- | --- | --- | --- |
| NAC-QD | 0 | 0 vs. 18 | 0.0366 | 3.225 | 0.015 |
| NAC-QD | 500 | 0 vs. 9 | 0.0346 | 3.051 | 0.027 |
| NAC-QD | 500 | 0 vs. 14 | 0.0432 | 3.808 | 0.002 |
| NAC-QD | 500 | 0 vs. 18 | 0.0445 | 3.921 | 0.001 |
| NAC-QD | 1000 | 0 vs. 6 | 0.0354 | 3.119 | 0.021 |
| NAC-QD | 1000 | 0 vs. 9 | 0.0386 | 3.403 | 0.008 |
| NAC-QD | 1000 | 0 vs. 14 | 0.043 | 3.786 | 0.002 |
| NAC-QD | 1000 | 0 vs. 18 | 0.045 | 3.962 | 0.001 |
| NAC-QD | 2500 | 0 vs. 14 | 0.039 | 3.436 | 0.007 |
| NAC-QD | 2500 | 0 vs. 18 | 0.0418 | 3.685 | 0.003 |
| NAC-QD | 5000 | 0 vs. 6 | 0.0326 | 2.868 | 0.047 |
| NAC-QD | 5000 | 0 vs. 9 | 0.0346 | 3.046 | 0.027 |
| NAC-QD | 5000 | 0 vs. 14 | 0.0407 | 3.588 | 0.004 |
| NAC-QD | 5000 | 0 vs. 18 | 0.043 | 3.788 | 0.002 |
| NAC-QD | 7500 | 0 vs. 14 | 0.0357 | 3.147 | 0.02 |
| NAC-QD | 7500 | 0 vs. 18 | 0.0387 | 3.41 | 0.008 |
| NAC-QD | 20000 | 0 vs. 14 | 0.0536 | 4.727 | <0.001 |
| NAC-QD | 20000 | 0 vs. 18 | 0.0682 | 6.011 | <0.001 |
| NAC-QD | 30000 | 0 vs. 14 | 0.0429 | 3.784 | 0.002 |
| NAC-QD | 30000 | 0 vs. 18 | 0.0553 | 4.87 | <0.001 |
| MPA-QD | 50 | 0 vs. 6 | 0.164 | 5.385 | <0.001 |
| MPA-QD | 50 | 0 vs. 9 | 0.166 | 5.458 | <0.001 |
| MPA-QD | 50 | 0 vs. 14 | 0.171 | 5.623 | <0.001 |
| MPA-QD | 50 | 0 vs. 18 | 0.185 | 6.079 | <0.001 |
| MPA-QD | 100 | 0 vs. 6 | 0.173 | 5.695 | <0.001 |
| MPA-QD | 100 | 0 vs. 9 | 0.176 | 5.793 | <0.001 |
| MPA-QD | 100 | 0 vs. 14 | 0.183 | 6.009 | <0.001 |
| MPA-QD | 100 | 0 vs. 18 | 0.185 | 6.08 | <0.001 |
| MPA-QD | 500 | 0 vs. 6 | 0.146 | 4.8 | <0.001 |
| MPA-QD | 500 | 0 vs. 9 | 0.148 | 4.86 | <0.001 |
| MPA-QD | 500 | 0 vs. 14 | 0.149 | 4.897 | <0.001 |
| MPA-QD | 500 | 0 vs. 18 | 0.168 | 5.531 | <0.001 |
| MPA-QD | 1000 | 0 vs. 6 | 0.105 | 3.462 | 0.007 |
| MPA-QD | 1000 | 0 vs. 9 | 0.105 | 3.468 | 0.007 |
| MPA-QD | 1000 | 0 vs. 14 | 0.111 | 3.641 | 0.004 |
| MPA-QD | 1000 | 0 vs. 18 | 0.127 | 4.166 | <0.001 |
| MPA-QD | 2500 | 0 vs. 6 | 0.138 | 4.53 | <0.001 |
| MPA-QD | 2500 | 0 vs. 9 | 0.14 | 4.588 | <0.001 |
| MPA-QD | 2500 | 0 vs. 14 | 0.143 | 4.697 | <0.001 |
| MPA-QD | 2500 | 0 vs. 18 | 0.138 | 4.553 | <0.001 |
| MPA-QD | 5000 | 0 vs. 6 | 0.136 | 4.47 | <0.001 |
| MPA-QD | 5000 | 0 vs. 9 | 0.137 | 4.502 | <0.001 |
| MPA-QD | 5000 | 0 vs. 14 | 0.143 | 4.692 | <0.001 |
| MPA-QD | 5000 | 0 vs. 18 | 0.145 | 4.78 | <0.001 |
| MPA-QD | 7500 | 0 vs. 6 | 0.13 | 4.276 | <0.001 |
| MPA-QD | 7500 | 0 vs. 9 | 0.132 | 4.346 | <0.001 |
| MPA-QD | 7500 | 0 vs. 14 | 0.136 | 4.479 | <0.001 |
| MPA-QD | 7500 | 0 vs. 18 | 0.149 | 4.894 | <0.001 |
| MPA-QD | 10000 | 0 vs. 6 | 0.203 | 6.68 | <0.001 |
| MPA-QD | 10000 | 0 vs. 9 | 0.205 | 6.751 | <0.001 |
| MPA-QD | 10000 | 0 vs. 14 | 0.213 | 7.002 | <0.001 |
| MPA-QD | 10000 | 0 vs. 18 | 0.221 | 7.28 | <0.001 |
| MPA-QD | 20000 | 0 vs. 18 | 0.0881 | 2.898 | 0.043 |
| CdCl_2_ | 0 | 0 vs. 9 | 0.109 | 3.235 | 0.015 |
| CdCl_2_ | 0 | 0 vs. 14 | 0.172 | 5.1 | <0.001 |
| CdCl_2_ | 0 | 0 vs. 18 | 0.221 | 6.538 | <0.001 |
| CdCl_2_ | 50 | 0 vs. 18 | 0.0997 | 2.951 | 0.036 |
| CdCl_2_ | 100 | 0 vs. 14 | 0.109 | 3.227 | 0.015 |
| CdCl_2_ | 100 | 0 vs. 18 | 0.128 | 3.799 | 0.002 |
| CdCl_2_ | 500 | 0 vs. 18 | 0.1 | 2.962 | 0.035 |
| CdCl_2_ | 1000 | 0 vs. 18 | 0.106 | 3.15 | 0.019 |
| CdCl_2_ | 5000 | 0 vs. 18 | 0.0987 | 2.922 | 0.04 |
| CdCl_2_ | 7500 | 0 vs. 18 | 0.109 | 3.221 | 0.015 |
| CdCl_2_ | 10000 | 0 vs. 18 | 0.107 | 3.18 | 0.018 |
| CdCl_2_ | 20000 | 0 vs. 14 | 0.127 | 3.759 | 0.002 |
| CdCl_2_ | 20000 | 0 vs. 18 | 0.173 | 5.108 | <0.001 |
| CdCl_2_ | 30000 | 0 vs. 9 | 0.101 | 3.003 | 0.031 |
| CdCl_2_ | 30000 | 0 vs. 14 | 0.152 | 4.496 | <0.001 |
| CdCl_2_ | 30000 | 0 vs. 18 | 0.206 | 6.103 | <0.001 |

Tab. S 4: Results from two-way ANOVA on quadruplicate absorbance data (597 nm) *P. velutina* incubated with NAC-QDs, MPA-QDs, and CdCl_2_ using timepoints and concentration. For pairwise t-test the Bonferroni post-hoc test was used. Only significant comparisons with p<0.05 are shown.

| Type | C/ µg l^-1^ | Comp. | Diff. of means | t | p |
| --- | --- | --- | --- | --- | --- |
| NAC-QD | 0 | 0 vs. 14 | 0.273 | 3.685 | 0.003 |
| NAC-QD | 0 | 0 vs. 18 | 0.31 | 4.18 | <0.001 |
| NAC-QD | 0 | 6 vs. 14 | 0.222 | 2.996 | 0.032 |
| NAC-QD | 0 | 6 vs. 18 | 0.259 | 3.492 | 0.006 |
| NAC-QD | 50 | 0 vs. 18 | 0.224 | 3.019 | 0.029 |
| NAC-QD | 500 | 0 vs. 14 | 0.329 | 4.445 | <0.001 |
| NAC-QD | 500 | 0 vs. 18 | 0.394 | 5.319 | <0.001 |
| NAC-QD | 500 | 6 vs. 14 | 0.223 | 3.011 | 0.03 |
| NAC-QD | 500 | 6 vs. 18 | 0.288 | 3.885 | 0.001 |
| NAC-QD | 1000 | 0 vs. 14 | 0.258 | 3.486 | 0.006 |
| NAC-QD | 1000 | 0 vs. 18 | 0.3 | 4.056 | <0.001 |
| NAC-QD | 2500 | 0 vs. 14 | 0.296 | 3.993 | <0.001 |
| NAC-QD | 2500 | 0 vs. 18 | 0.354 | 4.782 | <0.001 |
| NAC-QD | 2500 | 6 vs. 18 | 0.264 | 3.562 | 0.005 |
| NAC-QD | 5000 | 0 vs. 9 | 0.222 | 3.004 | 0.031 |
| NAC-QD | 5000 | 0 vs. 14 | 0.399 | 5.394 | <0.001 |
| NAC-QD | 5000 | 0 vs. 18 | 0.453 | 6.117 | <0.001 |
| NAC-QD | 5000 | 6 vs. 14 | 0.258 | 3.485 | 0.006 |
| NAC-QD | 5000 | 6 vs. 18 | 0.312 | 4.208 | <0.001 |
| NAC-QD | 5000 | 9 vs. 18 | 0.231 | 3.113 | 0.022 |
| NAC-QD | 7500 | 0 vs. 14 | 0.279 | 3.77 | 0.002 |
| NAC-QD | 7500 | 0 vs. 18 | 0.276 | 3.729 | 0.003 |
| NAC-QD | 10000 | 0 vs. 14 | 0.219 | 2.958 | 0.036 |
| NAC-QD | 10000 | 0 vs. 18 | 0.245 | 3.309 | 0.012 |
| NAC-QD | 10000 | 6 vs. 18 | 0.218 | 2.949 | 0.036 |
| MPA-QD | 0 | 0 vs. 14 | 0.143 | 3.467 | 0.007 |
| MPA-QD | 0 | 0 vs. 18 | 0.17 | 4.124 | <0.001 |
| MPA-QD | 0 | 6 vs. 14 | 0.175 | 4.227 | <0.001 |
| MPA-QD | 0 | 6 vs. 18 | 0.202 | 4.884 | <0.001 |
| MPA-QD | 0 | 9 vs. 18 | 0.157 | 3.796 | 0.002 |
| MPA-QD | 50 | 0 vs. 14 | 0.203 | 4.926 | <0.001 |
| MPA-QD | 50 | 0 vs. 18 | 0.25 | 6.06 | <0.001 |
| MPA-QD | 50 | 6 vs. 14 | 0.161 | 3.909 | 0.001 |
| MPA-QD | 50 | 6 vs. 18 | 0.208 | 5.043 | <0.001 |
| MPA-QD | 50 | 9 vs. 18 | 0.16 | 3.88 | 0.002 |
| MPA-QD | 100 | 0 vs. 14 | 0.14 | 3.393 | 0.009 |
| MPA-QD | 100 | 0 vs. 18 | 0.153 | 3.713 | 0.003 |
| MPA-QD | 100 | 6 vs. 14 | 0.152 | 3.681 | 0.003 |
| MPA-QD | 100 | 6 vs. 18 | 0.165 | 4.002 | <0.001 |
| MPA-QD | 500 | 0 vs. 14 | 0.213 | 5.154 | <0.001 |
| MPA-QD | 500 | 0 vs. 18 | 0.247 | 5.986 | <0.001 |
| MPA-QD | 500 | 6 vs. 14 | 0.166 | 4.012 | <0.001 |
| MPA-QD | 500 | 6 vs. 18 | 0.2 | 4.844 | <0.001 |
| MPA-QD | 500 | 9 vs. 14 | 0.133 | 3.212 | 0.016 |
| MPA-QD | 500 | 9 vs. 18 | 0.167 | 4.044 | <0.001 |
| MPA-QD | 1000 | 0 vs. 14 | 0.222 | 5.377 | <0.001 |
| MPA-QD | 1000 | 0 vs. 18 | 0.262 | 6.349 | <0.001 |
| MPA-QD | 1000 | 6 vs. 14 | 0.171 | 4.147 | <0.001 |
| MPA-QD | 1000 | 6 vs. 18 | 0.211 | 5.118 | <0.001 |
| MPA-QD | 1000 | 9 vs. 14 | 0.137 | 3.315 | 0.011 |
| MPA-QD | 1000 | 9 vs. 18 | 0.177 | 4.287 | <0.001 |
| MPA-QD | 2500 | 0 vs. 14 | 0.226 | 5.462 | <0.001 |
| MPA-QD | 2500 | 0 vs. 18 | 0.267 | 6.47 | <0.001 |
| MPA-QD | 2500 | 6 vs. 14 | 0.137 | 3.313 | 0.011 |
| MPA-QD | 2500 | 6 vs. 18 | 0.179 | 4.321 | <0.001 |
| MPA-QD | 2500 | 9 vs. 18 | 0.156 | 3.773 | 0.002 |
| MPA-QD | 5000 | 0 vs. 14 | 0.193 | 4.67 | <0.001 |
| MPA-QD | 5000 | 0 vs. 18 | 0.248 | 6 | <0.001 |
| MPA-QD | 5000 | 6 vs. 14 | 0.135 | 3.262 | 0.013 |
| MPA-QD | 5000 | 6 vs. 18 | 0.19 | 4.592 | <0.001 |
| MPA-QD | 5000 | 9 vs. 18 | 0.138 | 3.349 | 0.01 |
| MPA-QD | 7500 | 0 vs. 14 | 0.21 | 5.08 | <0.001 |
| MPA-QD | 7500 | 0 vs. 18 | 0.251 | 6.075 | <0.001 |
| MPA-QD | 7500 | 6 vs. 14 | 0.143 | 3.472 | 0.007 |
| MPA-QD | 7500 | 6 vs. 18 | 0.185 | 4.467 | <0.001 |
| MPA-QD | 7500 | 9 vs. 18 | 0.158 | 3.822 | 0.002 |
| MPA-QD | 10000 | 0 vs. 14 | 0.176 | 4.25 | <0.001 |
| MPA-QD | 10000 | 0 vs. 18 | 0.158 | 3.824 | 0.002 |
| MPA-QD | 10000 | 6 vs. 14 | 0.192 | 4.644 | <0.001 |
| MPA-QD | 10000 | 6 vs. 18 | 0.174 | 4.218 | <0.001 |
| MPA-QD | 10000 | 9 vs. 14 | 0.145 | 3.506 | 0.006 |
| MPA-QD | 10000 | 9 vs. 18 | 0.127 | 3.08 | 0.024 |
| CdCl_2_ | 0 | 0 vs. 14 | 0.298 | 5.771 | <0.001 |
| CdCl_2_ | 0 | 0 vs. 18 | 0.469 | 9.071 | <0.001 |
| CdCl_2_ | 0 | 6 vs. 14 | 0.305 | 5.899 | <0.001 |
| CdCl_2_ | 0 | 6 vs. 18 | 0.475 | 9.198 | <0.001 |
| CdCl_2_ | 0 | 9 vs. 14 | 0.205 | 3.961 | 0.001 |
| CdCl_2_ | 0 | 9 vs. 18 | 0.375 | 7.26 | <0.001 |
| CdCl_2_ | 0 | 14 vs. 18 | 0.17 | 3.299 | 0.012 |
| CdCl_2_ | 50 | 0 vs. 14 | 0.215 | 4.159 | <0.001 |
| CdCl_2_ | 50 | 0 vs. 18 | 0.385 | 7.461 | <0.001 |
| CdCl_2_ | 50 | 6 vs. 14 | 0.276 | 5.35 | <0.001 |
| CdCl_2_ | 50 | 6 vs. 18 | 0.447 | 8.652 | <0.001 |
| CdCl_2_ | 50 | 9 vs. 14 | 0.182 | 3.532 | 0.005 |
| CdCl_2_ | 50 | 9 vs. 18 | 0.353 | 6.834 | <0.001 |
| CdCl_2_ | 50 | 14 vs. 18 | 0.171 | 3.302 | 0.012 |
| CdCl_2_ | 100 | 0 vs. 14 | 0.156 | 3.021 | 0.029 |
| CdCl_2_ | 100 | 0 vs. 18 | 0.299 | 5.784 | <0.001 |
| CdCl_2_ | 100 | 6 vs. 14 | 0.249 | 4.815 | <0.001 |
| CdCl_2_ | 100 | 6 vs. 18 | 0.391 | 7.577 | <0.001 |
| CdCl_2_ | 100 | 9 vs. 14 | 0.154 | 2.988 | 0.032 |
| CdCl_2_ | 100 | 9 vs. 18 | 0.297 | 5.751 | <0.001 |
| CdCl_2_ | 500 | 0 vs. 14 | 0.182 | 3.528 | 0.005 |
| CdCl_2_ | 500 | 0 vs. 18 | 0.317 | 6.136 | <0.001 |
| CdCl_2_ | 500 | 6 vs. 14 | 0.261 | 5.053 | <0.001 |
| CdCl_2_ | 500 | 6 vs. 18 | 0.396 | 7.662 | <0.001 |
| CdCl_2_ | 500 | 9 vs. 14 | 0.172 | 3.335 | 0.011 |
| CdCl_2_ | 500 | 9 vs. 18 | 0.307 | 5.943 | <0.001 |
| CdCl_2_ | 1000 | 0 vs. 18 | 0.19 | 3.672 | 0.003 |
| CdCl_2_ | 1000 | 6 vs. 18 | 0.309 | 5.981 | <0.001 |
| CdCl_2_ | 1000 | 9 vs. 18 | 0.331 | 6.408 | <0.001 |
| CdCl_2_ | 1000 | 14 vs. 18 | 0.253 | 4.901 | <0.001 |
| CdCl_2_ | 2500 | 0 vs. 18 | 0.388 | 7.501 | <0.001 |
| CdCl_2_ | 2500 | 6 vs. 18 | 0.47 | 9.093 | <0.001 |
| CdCl_2_ | 2500 | 9 vs. 18 | 0.456 | 8.831 | <0.001 |
| CdCl_2_ | 2500 | 14 vs. 18 | 0.359 | 6.951 | <0.001 |
| CdCl_2_ | 30000 | 0 vs. 9 | 0.149 | 2.883 | 0.045 |

Tab. S 5: Results from two-way ANOVA on quadruplicate absorbance data (597 nm) of NAC-QDs, MPA-QDs, and CdCl_2_ in pure culture medium containing RB5 using timepoints and concentration. For pairwise t-test the Bonferroni post-hoc test was used. Only significant comparisons with p<0.05 are shown.

| Type | C/ µg l^-1^ | Comp. | Diff. of means | t | p |
| --- | --- | --- | --- | --- | --- |
| NAC-QD | 0 | 0 vs. 6 | 0.169 | 3.695 | 0.003 |
| NAC-QD | 0 | 0 vs. 9 | 0.352 | 7.68 | <0.001 |
| NAC-QD | 0 | 0 vs. 14 | 0.37 | 8.072 | <0.001 |
| NAC-QD | 0 | 0 vs. 18 | 0.35 | 7.655 | <0.001 |
| NAC-QD | 0 | 6 vs. 9 | 0.182 | 3.984 | 0.001 |
| NAC-QD | 0 | 6 vs. 14 | 0.2 | 4.377 | <0.001 |
| NAC-QD | 0 | 6 vs. 18 | 0.181 | 3.959 | 0.001 |
| NAC-QD | 50 | 0 vs. 9 | 0.359 | 7.85 | <0.001 |
| NAC-QD | 50 | 0 vs. 14 | 0.341 | 7.443 | <0.001 |
| NAC-QD | 50 | 0 vs. 18 | 0.34 | 7.424 | <0.001 |
| NAC-QD | 50 | 6 vs. 9 | 0.242 | 5.289 | <0.001 |
| NAC-QD | 50 | 6 vs. 14 | 0.224 | 4.882 | <0.001 |
| NAC-QD | 50 | 6 vs. 18 | 0.223 | 4.863 | <0.001 |
| NAC-QD | 100 | 0 vs. 9 | 0.348 | 7.599 | <0.001 |
| NAC-QD | 100 | 0 vs. 14 | 0.395 | 8.629 | <0.001 |
| NAC-QD | 100 | 0 vs. 18 | 0.368 | 8.042 | <0.001 |
| NAC-QD | 100 | 6 vs. 9 | 0.225 | 4.916 | <0.001 |
| NAC-QD | 100 | 6 vs. 14 | 0.272 | 5.946 | <0.001 |
| NAC-QD | 100 | 6 vs. 18 | 0.245 | 5.359 | <0.001 |
| NAC-QD | 500 | 0 vs. 6 | 0.205 | 4.476 | <0.001 |
| NAC-QD | 500 | 0 vs. 9 | 0.483 | 10.56 | <0.001 |
| NAC-QD | 500 | 0 vs. 14 | 0.551 | 12.034 | <0.001 |
| NAC-QD | 500 | 0 vs. 18 | 0.563 | 12.308 | <0.001 |
| NAC-QD | 500 | 6 vs. 9 | 0.279 | 6.083 | <0.001 |
| NAC-QD | 500 | 6 vs. 14 | 0.346 | 7.558 | <0.001 |
| NAC-QD | 500 | 6 vs. 18 | 0.359 | 7.831 | <0.001 |
| NAC-QD | 1000 | 0 vs. 6 | 0.169 | 3.694 | 0.003 |
| NAC-QD | 1000 | 0 vs. 9 | 0.35 | 7.641 | <0.001 |
| NAC-QD | 1000 | 0 vs. 14 | 0.492 | 10.758 | <0.001 |
| NAC-QD | 1000 | 0 vs. 18 | 0.479 | 10.454 | <0.001 |
| NAC-QD | 1000 | 6 vs. 9 | 0.181 | 3.948 | 0.001 |
| NAC-QD | 1000 | 6 vs. 14 | 0.323 | 7.064 | <0.001 |
| NAC-QD | 1000 | 6 vs. 18 | 0.309 | 6.76 | <0.001 |
| NAC-QD | 1000 | 9 vs. 14 | 0.143 | 3.117 | 0.022 |
| NAC-QD | 2500 | 0 vs. 9 | 0.29 | 6.335 | <0.001 |
| NAC-QD | 2500 | 0 vs. 14 | 0.497 | 10.858 | <0.001 |
| NAC-QD | 2500 | 0 vs. 18 | 0.511 | 11.161 | <0.001 |
| NAC-QD | 2500 | 6 vs. 9 | 0.19 | 4.155 | <0.001 |
| NAC-QD | 2500 | 6 vs. 14 | 0.397 | 8.678 | <0.001 |
| NAC-QD | 2500 | 6 vs. 18 | 0.411 | 8.982 | <0.001 |
| NAC-QD | 2500 | 9 vs. 14 | 0.207 | 4.523 | <0.001 |
| NAC-QD | 2500 | 9 vs. 18 | 0.221 | 4.827 | <0.001 |
| NAC-QD | 5000 | 0 vs. 6 | 0.143 | 3.118 | 0.022 |
| NAC-QD | 5000 | 0 vs. 9 | 0.273 | 5.955 | <0.001 |
| NAC-QD | 5000 | 0 vs. 14 | 0.557 | 12.162 | <0.001 |
| NAC-QD | 5000 | 0 vs. 18 | 0.551 | 12.036 | <0.001 |
| NAC-QD | 5000 | 6 vs. 14 | 0.414 | 9.044 | <0.001 |
| NAC-QD | 5000 | 6 vs. 18 | 0.408 | 8.918 | <0.001 |
| NAC-QD | 5000 | 9 vs. 14 | 0.284 | 6.207 | <0.001 |
| NAC-QD | 5000 | 9 vs. 18 | 0.278 | 6.081 | <0.001 |
| NAC-QD | 7500 | 0 vs. 14 | 0.391 | 8.55 | <0.001 |
| NAC-QD | 7500 | 0 vs. 18 | 0.357 | 7.806 | <0.001 |
| NAC-QD | 7500 | 6 vs. 14 | 0.372 | 8.133 | <0.001 |
| NAC-QD | 7500 | 6 vs. 18 | 0.338 | 7.39 | <0.001 |
| NAC-QD | 7500 | 9 vs. 14 | 0.356 | 7.78 | <0.001 |
| NAC-QD | 7500 | 9 vs. 18 | 0.322 | 7.036 | <0.001 |
| NAC-QD | 10000 | 0 vs. 14 | 0.256 | 5.6 | <0.001 |
| NAC-QD | 10000 | 0 vs. 18 | 0.32 | 6.992 | <0.001 |
| NAC-QD | 10000 | 6 vs. 14 | 0.283 | 6.187 | <0.001 |
| NAC-QD | 10000 | 6 vs. 18 | 0.347 | 7.579 | <0.001 |
| NAC-QD | 10000 | 9 vs. 14 | 0.267 | 5.824 | <0.001 |
| NAC-QD | 10000 | 9 vs. 18 | 0.33 | 7.216 | <0.001 |
| NAC-QD | 20000 | 9 vs. 18 | 0.14 | 3.054 | 0.026 |
| MPA-QD | 0 | 0 vs. 9 | 0.265 | 7.748 | <0.001 |
| MPA-QD | 0 | 0 vs. 14 | 0.331 | 9.668 | <0.001 |
| MPA-QD | 0 | 0 vs. 18 | 0.32 | 9.336 | <0.001 |
| MPA-QD | 0 | 6 vs. 9 | 0.222 | 6.477 | <0.001 |
| MPA-QD | 0 | 6 vs. 14 | 0.288 | 8.397 | <0.001 |
| MPA-QD | 0 | 6 vs. 18 | 0.276 | 8.064 | <0.001 |
| MPA-QD | 50 | 0 vs. 6 | 0.0998 | 2.913 | 0.041 |
| MPA-QD | 50 | 0 vs. 9 | 0.382 | 11.141 | <0.001 |
| MPA-QD | 50 | 0 vs. 14 | 0.47 | 13.724 | <0.001 |
| MPA-QD | 50 | 0 vs. 18 | 0.484 | 14.115 | <0.001 |
| MPA-QD | 50 | 6 vs. 9 | 0.282 | 8.228 | <0.001 |
| MPA-QD | 50 | 6 vs. 14 | 0.37 | 10.811 | <0.001 |
| MPA-QD | 50 | 6 vs. 18 | 0.384 | 11.202 | <0.001 |
| MPA-QD | 50 | 9 vs. 18 | 0.102 | 2.974 | 0.034 |
| MPA-QD | 100 | 0 vs. 9 | 0.265 | 7.749 | <0.001 |
| MPA-QD | 100 | 0 vs. 14 | 0.38 | 11.102 | <0.001 |
| MPA-QD | 100 | 0 vs. 18 | 0.364 | 10.639 | <0.001 |
| MPA-QD | 100 | 6 vs. 9 | 0.247 | 7.222 | <0.001 |
| MPA-QD | 100 | 6 vs. 14 | 0.362 | 10.574 | <0.001 |
| MPA-QD | 100 | 6 vs. 18 | 0.346 | 10.111 | <0.001 |
| MPA-QD | 100 | 9 vs. 14 | 0.115 | 3.352 | 0.01 |
| MPA-QD | 100 | 9 vs. 18 | 0.099 | 2.89 | 0.044 |
| MPA-QD | 500 | 0 vs. 9 | 0.185 | 5.399 | <0.001 |
| MPA-QD | 500 | 0 vs. 14 | 0.428 | 12.485 | <0.001 |
| MPA-QD | 500 | 0 vs. 18 | 0.431 | 12.568 | <0.001 |
| MPA-QD | 500 | 6 vs. 9 | 0.143 | 4.183 | <0.001 |
| MPA-QD | 500 | 6 vs. 14 | 0.386 | 11.269 | <0.001 |
| MPA-QD | 500 | 6 vs. 18 | 0.389 | 11.351 | <0.001 |
| MPA-QD | 500 | 9 vs. 14 | 0.243 | 7.086 | <0.001 |
| MPA-QD | 500 | 9 vs. 18 | 0.246 | 7.168 | <0.001 |
| MPA-QD | 1000 | 0 vs. 9 | 0.188 | 5.486 | <0.001 |
| MPA-QD | 1000 | 0 vs. 14 | 0.494 | 14.405 | <0.001 |
| MPA-QD | 1000 | 0 vs. 18 | 0.52 | 15.176 | <0.001 |
| MPA-QD | 1000 | 6 vs. 9 | 0.11 | 3.22 | 0.015 |
| MPA-QD | 1000 | 6 vs. 14 | 0.416 | 12.14 | <0.001 |
| MPA-QD | 1000 | 6 vs. 18 | 0.442 | 12.911 | <0.001 |
| MPA-QD | 1000 | 9 vs. 14 | 0.306 | 8.92 | <0.001 |
| MPA-QD | 1000 | 9 vs. 18 | 0.332 | 9.69 | <0.001 |
| MPA-QD | 2500 | 0 vs. 9 | 0.144 | 4.217 | <0.001 |
| MPA-QD | 2500 | 0 vs. 14 | 0.367 | 10.7 | <0.001 |
| MPA-QD | 2500 | 0 vs. 18 | 0.49 | 14.296 | <0.001 |
| MPA-QD | 2500 | 6 vs. 14 | 0.291 | 8.485 | <0.001 |
| MPA-QD | 2500 | 6 vs. 18 | 0.414 | 12.081 | <0.001 |
| MPA-QD | 2500 | 9 vs. 14 | 0.222 | 6.483 | <0.001 |
| MPA-QD | 2500 | 9 vs. 18 | 0.345 | 10.079 | <0.001 |
| MPA-QD | 2500 | 14 vs. 18 | 0.123 | 3.596 | 0.004 |
| MPA-QD | 5000 | 0 vs. 14 | 0.203 | 5.936 | <0.001 |
| MPA-QD | 5000 | 0 vs. 18 | 0.341 | 9.963 | <0.001 |
| MPA-QD | 5000 | 6 vs. 14 | 0.184 | 5.386 | <0.001 |
| MPA-QD | 5000 | 6 vs. 18 | 0.322 | 9.412 | <0.001 |
| MPA-QD | 5000 | 9 vs. 14 | 0.108 | 3.15 | 0.019 |
| MPA-QD | 5000 | 9 vs. 18 | 0.246 | 7.176 | <0.001 |
| MPA-QD | 5000 | 14 vs. 18 | 0.138 | 4.027 | <0.001 |
| MPA-QD | 7500 | 0 vs. 14 | 0.162 | 4.72 | <0.001 |
| MPA-QD | 7500 | 0 vs. 18 | 0.304 | 8.887 | <0.001 |
| MPA-QD | 7500 | 6 vs. 18 | 0.229 | 6.695 | <0.001 |
| MPA-QD | 7500 | 9 vs. 14 | 0.13 | 3.801 | 0.002 |
| MPA-QD | 7500 | 9 vs. 18 | 0.273 | 7.967 | <0.001 |
| MPA-QD | 7500 | 14 vs. 18 | 0.143 | 4.167 | <0.001 |
| MPA-QD | 10000 | 9 vs. 14 | 0.122 | 3.571 | 0.005 |
| CdCl_2_ | 0 | 0 vs. 9 | 0.277 | 4.773 | <0.001 |
| CdCl_2_ | 0 | 0 vs. 14 | 0.989 | 17.031 | <0.001 |
| CdCl_2_ | 0 | 0 vs. 18 | 1.089 | 18.745 | <0.001 |
| CdCl_2_ | 0 | 6 vs. 9 | 0.217 | 3.731 | 0.003 |
| CdCl_2_ | 0 | 6 vs. 14 | 0.929 | 15.989 | <0.001 |
| CdCl_2_ | 0 | 6 vs. 18 | 1.028 | 17.704 | <0.001 |
| CdCl_2_ | 0 | 9 vs. 14 | 0.712 | 12.258 | <0.001 |
| CdCl_2_ | 0 | 9 vs. 18 | 0.812 | 13.972 | <0.001 |
| CdCl_2_ | 50 | 0 vs. 14 | 0.833 | 14.343 | <0.001 |
| CdCl_2_ | 50 | 0 vs. 18 | 0.842 | 14.496 | <0.001 |
| CdCl_2_ | 50 | 6 vs. 14 | 0.866 | 14.91 | <0.001 |
| CdCl_2_ | 50 | 6 vs. 18 | 0.875 | 15.064 | <0.001 |
| CdCl_2_ | 50 | 9 vs. 14 | 0.781 | 13.443 | <0.001 |
| CdCl_2_ | 50 | 9 vs. 18 | 0.79 | 13.596 | <0.001 |
| CdCl_2_ | 100 | 0 vs. 14 | 0.617 | 10.626 | <0.001 |
| CdCl_2_ | 100 | 0 vs. 18 | 0.675 | 11.613 | <0.001 |
| CdCl_2_ | 100 | 6 vs. 14 | 0.681 | 11.72 | <0.001 |
| CdCl_2_ | 100 | 6 vs. 18 | 0.738 | 12.707 | <0.001 |
| CdCl_2_ | 100 | 9 vs. 14 | 0.529 | 9.106 | <0.001 |
| CdCl_2_ | 100 | 9 vs. 18 | 0.586 | 10.094 | <0.001 |
| CdCl_2_ | 500 | 0 vs. 14 | 0.561 | 9.655 | <0.001 |
| CdCl_2_ | 500 | 0 vs. 18 | 0.764 | 13.145 | <0.001 |
| CdCl_2_ | 500 | 6 vs. 14 | 0.621 | 10.683 | <0.001 |
| CdCl_2_ | 500 | 6 vs. 18 | 0.823 | 14.173 | <0.001 |
| CdCl_2_ | 500 | 9 vs. 14 | 0.604 | 10.401 | <0.001 |
| CdCl_2_ | 500 | 9 vs. 18 | 0.807 | 13.891 | <0.001 |
| CdCl_2_ | 500 | 14 vs. 18 | 0.203 | 3.49 | 0.006 |
| CdCl_2_ | 1000 | 0 vs. 18 | 0.518 | 8.917 | <0.001 |
| CdCl_2_ | 1000 | 6 vs. 18 | 0.608 | 10.459 | <0.001 |
| CdCl_2_ | 1000 | 9 vs. 18 | 0.562 | 9.673 | <0.001 |
| CdCl_2_ | 1000 | 14 vs. 18 | 0.513 | 8.838 | <0.001 |
| CdCl_2_ | 2500 | 0 vs. 18 | 0.632 | 10.87 | <0.001 |
| CdCl_2_ | 2500 | 6 vs. 18 | 0.698 | 12.018 | <0.001 |
| CdCl_2_ | 2500 | 9 vs. 18 | 0.661 | 11.383 | <0.001 |
| CdCl_2_ | 2500 | 14 vs. 18 | 0.584 | 10.052 | <0.001 |

Tab. S 6: Parameters calculated from the 4 parametric logistic curves (Hill’s type) for experiments with NAC-QDs, MPA-QDs, and CdCl_2_ with *P. velutina* and without RB5. p represents the p-value

| type | time/ d | EC50/ µg l^-1^ | min/ µg l^-1^ | max/ µg l^-1^ | Hillslope | R² | p(EC50) | p(min) | p(max) | p(Hillslope) |
| --- | --- | --- | --- | --- | --- | --- | --- | --- | --- | --- |
| NAC-QD | 0 | 1.0806E-17 | 0.1012 | 0.1271 | 11699.4 | 0.0052 | 1 | 1 | 1 | 1 |
|  | 6 | 9.4293E-13 | 0.1522 | 0.1889 | 3.462 | 0.0069 | 1 | 1 | 1 | 1 |
|  | 9 | 13827.8206 | 0.0223 | 0.2869 | -5.0173 | 0.4722 | 0.0039 | 0.7479 | <0.0001 | 0.2342 |
|  | 14 | 17222.2406 | 0.0228 | 0.4144 | -6.6161 | 0.5241 | <0.0001 | 0.7999 | <0.0001 | 0.5238 |
|  | 18 | 17575.3995 | -0.0029 | 0.4578 | -4.8815 | 0.5392 | <0.0001 | 0.982 | <0.0001 | 0.2832 |
| MPA-QD | 0 | 57065.8796 | 0.0434 | 0.0445 | 356726 | -6.6613E-16 | 1 | 1 | 1 | 1 |
|  | 6 | 0.0003 | 0.0683 | 0.0689 | 4.2282 | 0.000012439 | 1 | 1 | 1 | 1 |
|  | 9 | 9014.8296 | 0.0339 | 0.1291 | -9.455 | 0.4752 | <0.0001 | 0.0295 | <0.0001 | 0.2186 |
|  | 14 | 12306.0651 | 0.0224 | 0.2443 | -7.1707 | 0.5969 | 0.007 | 0.5483 | <0.0001 | 0.5041 |
|  | 18 | 10628.1961 | 0.0249 | 0.282 | -9.0361 | 0.6425 | <0.0001 | 0.3743 | <0.0001 | 0.5218 |
| CdCl_2_ | 0 | 49.5479 | 0.0201 | 0.124 | 45.9778 | 0.1375 | 1 | 0.6233 | <0.0001 | 1 |
|  | 6 | 6287.7748 | 0.0099 | 0.0329 | -2416.65 | 0.1529 | 1 | 1 | 1 | 1 |
|  | 9 | 723.5979 | 0.0132 | 0.1264 | -7.8691 | 0.7365 | 0.0004 | 0.0607 | <0.0001 | 0.1915 |
|  | 14 | 1119.1142 | 0.0005 | 0.3151 | -1.473 | 0.7645 | 0.0019 | 0.9854 | <0.0001 | 0.0218 |
|  | 18 | 4453.4196 | 0.0096 | 0.4471 | -34.2991 | 0.8133 | 1 | 0.761 | <0.0001 | 1 |

Tab. S 7: Parameters calculated from the 4 parametric logistic curves (Hill’s type) for experiments with NAC-QDs, MPA-QDs, and CdCl_2_ with *P. velutina* and with RB5. p represents the p-value.

| type | time/ d | EC50/ µg l^-1^ | min/ µg l^-1^ | max/ µg l^-1^ | Hillslope | R² | p(EC50) | p(min) | p(max) | p(Hillslope) |
| --- | --- | --- | --- | --- | --- | --- | --- | --- | --- | --- |
| NAC-QD | 0 | 183.376 | 0.2666 | 0.2996 | -0.2686 | 0.0126 | 0.2282 | <0.0001 | 0.9844 | 0.9329 |
|  | 6 | 9775.868 | 0.1452 | 0.3054 | 5.411 | 0.6323 | <0.0001 | <0.0001 | <0.0001 | 0.1724 |
|  | 9 | 7262.1266 | -0.074 | 0.3484 | 3.2086 | 0.8742 | <0.0001 | <0.0001 | <0.0001 | 0.0013 |
|  | 14 | 10194.7048 | -0.1682 | 0.3127 | 65.9034 | 0.8062 | 1 | <0.0001 | <0.0001 | 1 |
|  | 18 | 14851.2403 | -0.1595 | 0.3421 | 6.7718 | 0.7396 | <0.0001 | <0.0001 | <0.0001 | 0.071 |
| MPA-QD | 0 | 98.6774 | 0.2949 | 0.3687 | 0.772 | 0.5367 | 0.1703 | <0.0001 | <0.0001 | 0.0954 |
|  | 6 | 2236.8887 | 0.2462 | 0.3715 | 0.4184 | 0.4137 | 0.812 | <0.0001 | 0.0005 | 0.2915 |
|  | 9 | 932.1613 | -0.0047 | 0.3643 | 0.9484 | 0.8746 | 0.0016 | 0.8375 | <0.0001 | 0.0009 |
|  | 14 | 4694.2421 | -0.0894 | 0.3387 | 2.1051 | 0.9036 | <0.0001 | <0.0001 | <0.0001 | 0.0002 |
|  | 18 | 8176.6606 | -0.0816 | 0.3725 | 7.759 | 0.8559 | <0.0001 | <0.0001 | <0.0001 | 0.0058 |
| CdCl_2_ | 0 | 49.435 | 0.8051 | 0.9246 | -26.1678 | 0.5337 | 0.9999 | <0.0001 | <0.0001 | 1 |
|  | 6 | 53615.8213 | -2693.3521 | 0.8801 | -20.01 | 0.0473 | 1 | 1 | <0.0001 | 1 |
|  | 9 | 45.8454 | 0.6498 | 0.8571 | 0.7545 | 0.5844 | 0.1462 | <0.0001 | <0.0001 | 0.1543 |
|  | 14 | 583.1797 | 0.0463 | 0.8232 | 6.1623 | 0.9362 | <0.0001 | 0.1022 | <0.0001 | 0.0382 |
|  | 18 | 2634.5596 | 0.0634 | 0.8129 | 34.6306 | 0.8229 | 1 | 0.1188 | <0.0001 | 1 |

Tab. S 8: Pearson linear correlation for decolorization vs. absorption increase.

| C/ µg l^-1^ | NAC-QDs | MPA-QDs | CdCl_2_ |
| --- | --- | --- | --- |
| 0 | -0.8682 | -0.8220 | -0.9729 |
| 50 | -0.8367 | -0.9186 | -0.9381 |
| 100 | -0.8107 | -0.9279 | -0.9500 |
| 500 | -0.9408 | -0.9844 | -0.9755 |
| 1000 | -0.9717 | -0.9945 | -0.9470 |
| 2500 | -0.9873 | -0.9829 | -0.9937 |
| 5000 | -0.9916 | -0.9730 | -0.9021 |
| 7500 | -0.9593 | -0.9289 | -0.8032 |
| 10000 | -0.9765 | -0.7892 | -0.8077 |
| 20000 | -0.3034 | -0.3011 | 0.2647 |
| 30000 | 0.0366 | 0.6665 | -0.4002 |
